# Vitreous Carbon, Geometry and Topology: A Hollistic Approach

**DOI:** 10.3390/nano11071694

**Published:** 2021-06-28

**Authors:** Patrice Mélinon

**Affiliations:** 1 Université de Lyon, F-69000 Lyon, France; patrice.melinon@univ-lyon1.fr; 2 Institut Lumière Matière, Université Claude Bernard Lyon 1, CEDEX, F69622 Villeurbanne, France

**Keywords:** vitreous carbon, thin films, applied topology

## Abstract

Glass-like carbon (*GLC*) is a complex structure with astonishing properties: isotropic $sp^{2}$ structure, low density and chemical robustness. Despite the expanded efforts to understand the structure, it remains little known. We review the different models and a physical route (pulsed laser deposition) based on a well controlled annealing of the native *2D*/*3D* amorphous films. The many models all have compromises: neither all bad nor entirely satisfactory. Properties are understood in a single framework given by topological and geometrical properties. To do this, we present the basic tools of topology and geometry at a ground level for *2D* surface, graphene being the best candidate to do this. With this in mind, special attention is paid to the hyperbolic geometry giving birth to triply periodic minimal surfaces. Such surfaces are the basic tools to understand the *GLC* network architecture. Using two theorems (the classification and the uniformisation), most of the *GLC* properties can be tackled at least at a heuristic level. All the properties presented can be extended to *2D* materials. It is hoped that some researchers may find it useful for their experiments.

## 1. Introduction

**Figure 1:**
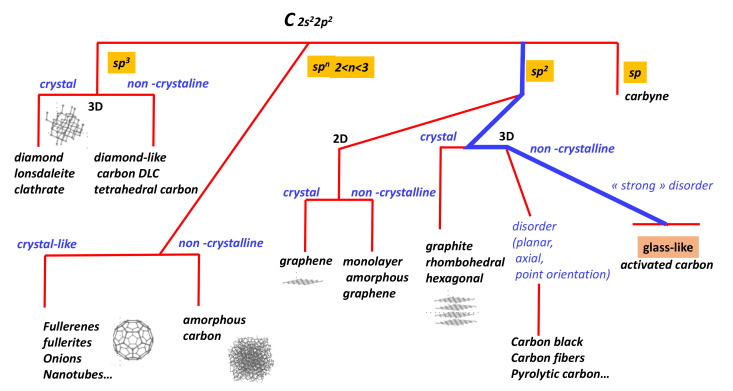
Landscape of the carbon structure according to hybridisation. The glassy carbon pathway is underlined in blue.

Carbon is the most versatile element of the periodic table. There are nearly ten million known carbon compounds, and an entire branch of chemistry, known as organic chemistry, is devoted to their study (see Figure 1). Many carbon compounds are essential for life as we know it. Even though the number of allotropes is lower than in boron or sulphur, carbon exists in several forms which are characterised by a simple but powerful tool introduced by chemists: hybridisation. This paper focuses on the special case of $sp^{2}$ hybridisation, which is planar. Graphene, which is the tessellation of a flat surface with hexagons, mimics the concept of surface in Euclidean space for mathematics. It is therefore legitimate to use the concepts related to geometry and surface topology [1] to understand the structure of some carbon allotropes, especially vitreous carbon. The so-called glass-like or vitreous carbon (*GLC*) [2] exhibits excellent biological compatibility with living tissues, meaning it has a high potential for applications in life science [3]. Moreover, glass-like carbon has interesting properties including “high temperature resistance”, hardness, low density, low electrical resistance, low friction, low thermal resistance, extreme resistance to chemical attack and impermeability to gases and liquids despite porosity [4] (the zero open porosity gives a low permeability to gases). The structure of glass-like carbon has long been a subject of debate. The main properties of *GLC* are summarised in Table 1. Surprisingly, despite huge works dedicated to all forms of carbon over the last decades (graphene, fullerenes, diamond, carbon foams, onions, nanotubes, clathrate, etc.), the *GLC* structure remains a bit of a mystery. Several hundred papers suggesting “new” carbon allotropes [5,6] have been published but no one is in a position to explain *GLC*. *GLC* no longer has a long-range crystal order. Disordered phases of carbon have always proved difficult to characterise as the standard structural determination methods based on diffraction are not relevant. The vitreous carbon is intimately related to the open issues of graphitising versus non-graphitising carbon.

**Figure 2:**
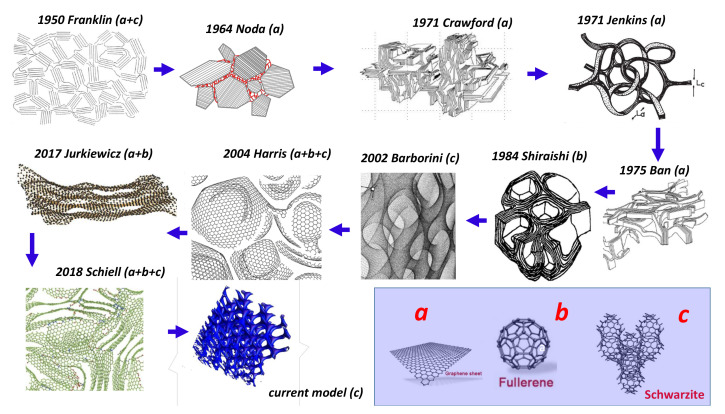
History and evolution of *GLC* throughout the ages, as presented by Franklin [7,8], Noda et al. [9], Crawford et al. [10], Jenkis et al. [12], Ban et al. [11], Shiraishi et al. [13], Barborini et al. [14], Harris [17], Jurkiewicz et al. [18] and Shiell et al. [20]. The insert (bottom right) shows the three elemental forms according to the curvature sign with labels a–c, respectively. The labels in the models correspond to the elemental bricks of the models. Figure 2 is adapted from [24]. Reproduced with permission from Shiell, Journal of Non-Crystalline Solids; copyright 2021, Elsevier.

The first model was proposed by Franklin [7,8] (see Figure 2). The keypoint is the existence of saddle points with negative curvature connecting graphitic regions. Noda and Inakagi proposed in 1964 [9] a structural model of *GLC* deduced from X-ray diffraction, in which tetrahedral carbon atoms form the main part of the cross-linkages which link the graphite-like layers in a random way. The weakness of the model is the large content of $sp^{3}$ hybridised atoms and the “high density”; it is better suited for isotropic carbon, which is a graphitisable material. Using electron microscopy, Crawford et al. [10] proposed a graphene (graphite) lattice distortion due to non-basal edge dislocations and low-angle boundaries of tilt or twist character. In the same way, the model of Ban et al. [11] consists of a high proportion of intertwined crystallites comprising turbostratically packed aggregates of graphitic basal planes. From electron microscopy, Jenkins and Kawamure [12] proposed a model based on aromatic ($sp^{2}$ hybridised carbon atoms) ribbon tangling. The weakness is the open pores inconsistent with gas impermeability and the presence of ribbon edges which are chemically active. In the “nested fullerene” model of Shiraishi [13], the form of the stacks of aromatic layers is isometric, not ribbon-like. Barborini et al. [14] synthesised a “spongy carbon” by energy cluster beam deposition. This is a distinctly different concept where the skeleton is derived from the *TPMS* (triply minimal surface structures) introduced by Townsed et al. [15]. The keypoint is that total energy calculations have shown that carbon Schwarzites are more stable than fullerenes [16]. Harris [17] proposed a model for the structure of non-graphitising carbons, which consists of fragments of curved carbon sheets (fullerene-like), containing pentagons and heptagons as well as hexagons. Thanks to neutron and X-ray diffraction, Jurkiewicz et al. [18] showed a large proportion of curved graphitic sheets. The presence of these curved elements in carbon nanomaterials can be related to the formation of topological point-type defects in non-hexagonal rings (pentagons, heptagons and higher-membered rings). Acharya et al. [19] developed an interesting model for nanoporous carbon based on an algorithm approach. This method starts from the basic building blocks and lets random bond formation between hexagonal sheets lead to curvature. n-fold rings are obtained by connecting unsaturated carbon atoms based on a minimum distance criterion. Shiell et al. [20] proposed a structure with graphene layers with different orientations. The last model, discussed below, is derived from the gyroid Schwarzite using a procedure given by Benedek et al. [21,22]. Other observations predict that carbon foams contain graphite-like “*sp*${}^{2}$ carbon” segments, connected by $sp^{3}$ carbon atoms, resulting in porous Kagome structures [23]. However, such networks are widely open structures and permeable to gases. Table 1 reports the main features of glass-like carbon. Samples obeying all theses features can be considered as *GLC*.

## 2. Characterisation of *GLC*

### 2.1. Raman Spectroscopy

*GLC* is improperly considered as a *3D* amorphous structure, but it avoids the dangling bonds formation as reported in all families of common *3D* amorphous carbon, which are formed by a mixing of $sp^{2}/sp^{3}$ and dangling bonds. *GLC* has the expected short-range order probed by Raman spectroscopy with two narrow bands labelled $I_{D}$ and $I_{G}$ with $I_{D}/I_{G}>1.5$ [32]. In addition to the $E_{2g}$ zone centre vibration in graphite $I_{G}$, another band labelled $I_{D}$ at 1350 cm${}^{-1}$ is disorder induced and can be assigned to scattering by off-centre phonons made active by a relaxation of the wavevector selection rules due to finite crystalline size [33]. The *G* peak is due to the bond stretching of all pairs of $sp^{2}$ atoms in rings, the *D* peak is thus due to the breathing modes of $sp^{2}$ atoms in rings. However, the complete analysis of the $I_{D}/I_{G}$ ratio and the $I_{G}$ band width remains elusive. Raman spectra presents some striking similarities with reduced graphene oxide [34] *RGO* excepted the weak ratio $I_{D}/I_{G}<1$ in *RGO*. Classical analysis of nanocrystalline graphite $I_{D}/I_{G}$∼$1/L_{a}$, $L_{a}$ being the $sp^{2}$ cluster size according to the Tuinstra–Koenig [35] model or in *3D* amorphous carbon $I_{D}/I_{G}$∼$L_{a}^{2}$ [33], is inconsistent with the expected size deduced from the $I_{D}/I_{G}$ ratio [33] and the *G* peak full width at half maximum (*FWHM*), which is a measure of disorder and increases continuously as the disorder increases [36]. In summary, large ratio $I_{D}/I_{G}$ is inconsistent with narrow *G* and *D* bands. However, the *G* mode does not require the presence of sixfold rings, and thus it occurs at all $sp^{2}$ sites, not only those in rings [33]. *GLC* cannot be transformed into crystalline graphite even at high temperatures (3000 ${}^{\circ }$C) and belongs to the class of “non-graphitising carbon” [37]. Thus, there is a consensus that *GLC* is isotropic and fully $sp^{2}$ hybridised. When it comes to this issue, it is clear that Raman spectroscopy reaches its limits. For example, *GLC* matches very well with the Raman spectrum of the graphite-tiled inverse opal. The carbon inverse opals strongly diffract visible light [38] and the lattice parameter (mesoscale) does not match with the nanoporous structure that characterises *GLC*. Pier et al. [39] reported carbon nanotubes with striking Raman similarities with *GLC*.

**Figure 3:**
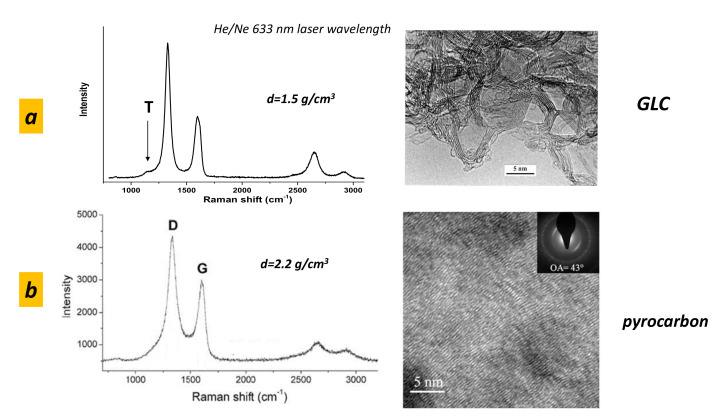
TEM and Raman observations: comparison between pyrocarbon [41] and *GLC* [26]: (**a**) GLC; and (**b**) pyrocarbon. The Raman features are close but HRTEM patterns are quite different (except *T* band and second-order features). To remove doubts about the structure, further spectroscopic tools are necessary. (**b**) Reproduced with permission from López-Honorato, Carbon [41]; copyright 2010, Elsevier.

The low-frequency region provides useful information. In the low-frequency region between 150 and 550 cm${}^{-1}$, two Raman peaks located at 224 and 334 cm${}^{-1}$ are observed as a signature of the curvature-related structures that are normally present in *GLC*. These peaks are also found for fullerenes, carbon nanotubes or carbon onion structures, but they are completely absent in the case of graphite [26]. This is the first observation of the “non-zero curvature” in non-graphitisable networks. To illustrate the ambiguity, Figure 3 shows some striking similarities between Raman bands observed in pyrocarbon [40] (graphitisable) and *GLC* (non-graphitisable).

### 2.2. Other Spectroscopies

Raman spectroscopy is a powerful tool for *GLC* analysis. Thus, there is no doubt that further investigations are needed. Transmission electron microscope (*TEM*) provides supplementary information that could be compared to simulation. The model proposed by Harris and Tsang [17,42] for microporous carbon produced by arc evaporation is based on curved sheets of graphite. The curvature is introduced by including pentagons and heptagons, together with hexagons, as starting fragments. In all graphitisable compounds, the intense electron irradiation in the microscope induces a transformation of the film with the formation of quasi-spherical concentric fullerene particles (carbon onions). This graphitisation is not observed in *GLC* and the stability of *GLC* under irradiation is one of the signatures of *GLC*. Note that a combustion route is proposed in which oxygen attacks the structural units that inhibit graphitisation [43]. The concentration of oxygen atoms in the film seems to be a key parameter. Unfortunately, poor data are available in the literature. Porous *3D*-graphene-based materiala [44] have a *TEM* structure close to *GLC*. These materials consist mainly of defected/wrinkled graphene sheets in the dimensional size of a few nanometers, with at least some covalent bonds between each other. Unfortunately, such samples used for supercapacitors are permeable and $I_{D}/I_{G}$ is lower than the expected value in *GLC*. *TEM* observations reveal that graphite foam [45] is a porous structure massively $sp^{2}$ hybridised regularly shaped but concave, while *GLC* is a more complex structure with a mixing of concave and convex curvature.

## 3. Synthesis of *GLC* Thin Films

### 3.1. Experimental Set Up

**Figure 4:**
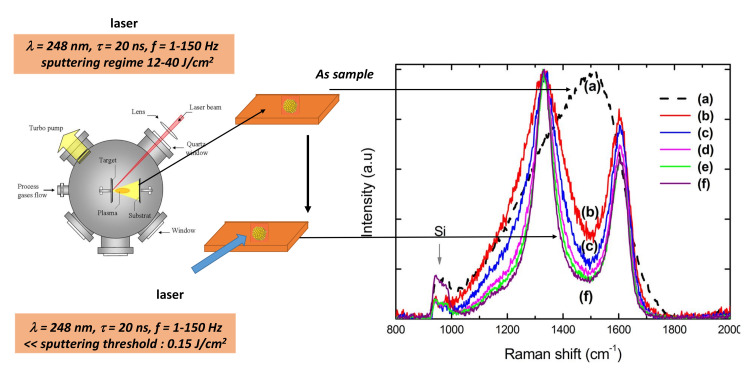
(**Left**) Synopsis of the *PLD* apparatus. (**Right**) Carbon thin film annealing. Raman features are compatible with a *3D* amorphous structure in the as-deposited film. After several laser shots, the film presents the features of *GLC* (corroborated by other spectroscopic tools, see below): (a) un-irradiated carbon; (b) 5 shots; (c) 10 shots; (d) 100 shots; (e) 200 shots; and (f) 1000 shots [46].

**Figure 5:**
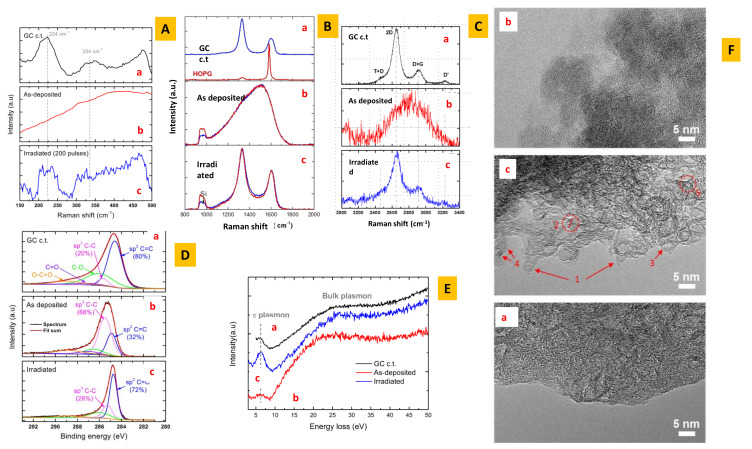
Selected properties of thin films of *GLC* compared to amorphous carbon and commercial glass-like carbon. The transformation of the *2D*/*3D* amorphous phase in *GLC* in the thin film can be studied in detail contrary to the massive pyrolysis process at high temperature ($3000^{\;\circ }$C). This is of prime importance to understanding the non-graphitisation mechanism. (a–c) are assigned to commercial glass-like carbon (GC c.t.), as-deposited sample and irradiated sample (1000 shots), respectively. (**A**–**C**) The three region in the Raman spectra (low-frequency, first-order and second-order spectra, respectively). The two Raman peaks located at 224 and 334 cm${}^{-1}$ (**A**) are the signature of curvature-related structures which are normally present in *GLC*. These peaks are also found for fullerenes, carbon nanotubes or carbon onion structures [49], but they are completely absent in the case of graphite [26]. The second-order Raman spectra (**C**) are symmetric and differ from graphitisable samples where asymmetry is observed [50]. (**D**) The core level lines in the $C_{1s}$ region observed in *XPS*. Note the high percentage of $sp^{3}$ hybridisation in the as-deposited sample. (**E**) The π–π plasmon band peak (merged with $\pi -\pi^{*}$ interband transition) that characterises a massively $sp^{2}$ structure in *GLC*. (**F**) The *TEM* observation showing the open structure with a “spaghetti tangle” in *GLC* (c). The figures are taken from [46]. Reproduced with permission from Diaf, Phys. Rev Mat.; copyright 2021, American Physical Society.

Details are given elsewhere [46]. Carbon thin films (in the broadest sense a film of thickness less than 100 nm) were deposited by pulsed laser deposition (*PLD*) at room temperature by means of a pulsed *KrF* excimer laser (Lambda Physik; λ = 248 nm; pulse duration τ = 20 ns; and repetition rate f = 10 Hz). The nature of the carbon target (graphite, *GLC*, etc.) is not relevant. Carbon is-deposited onto silicon substrates. Thin films (10–30 nm) are deposited onto Si. All the features are assigned to *2D*/*3D* amorphous carbon (see Figure 4). At this stage, the term *2D*/*3D* amorphous should be explained. *3D* amorphous carbon is assigned to the absence of long-range periodicity and a wide distribution of bond lengths, bond angles and hybridisations (namely, $sp^{2}/sp^{3}$). More recently, monolayers of $sp^{2}$-bonded amorphous carbon films have been synthesised [47,48]. Monolayer amorphous carbon reveals a wide distribution of bond lengths, bond angles and topological defects, but, contrary to *3D*, amorphous carbon hybridisation is largely $sp^{2}$. In our case, dealing with the film thickness, the geometry is intermediate between a macroscopic sample (*3D*) and a monolayer (*2D*). According to the electronic structure, the massively $sp^{2}$ hybridisation may suggest a striking similarity with $sp^{2}$-bonded amorphous carbon extended to a *3D* network. GLC is also different from three-dimensional aggregation of graphene which is not isotropic [48]. The *2D*/*3D* amorphous carbon layers were irradiated in air at the optimal fluence threshold of 0.15 J/cm${}^{2}$. The Raman features change with the number of laser shots (Figure 4). Figure 5 displays the optimal irradiation for the production of *GLC* carbon.

### 3.2. *GLC* Characterisation

Figure 5 summarises all the features that corroborate the thin film *GLC* structure. To guide the eye, the *GLC* films are compared with a commercial *GLC* sample obtained by high-temperature pyrolysis (see details in [46]). The presence of $sp^{3}$ precursors (see *XPS* spectra) in the as-deposited sample ensures a non-graphitisation route during laser annealing. This is the keypoint for the *GLC* synthesis and is discussed below.

## 4. Topology/Geometry

At first glance, *TEM* image reveals for all *GLC* some common features: graphitic curved, tangled and twisted sheets or ribbons with saddle points (negative curvature) connecting graphitic regions. Each property could be discussed in terms of “topology”.

### 4.1. Algebraic Topology VERSUS Geometry: From Mathematics to Physics

In 2017, David Castelvecchi [51] reported in a paper a citation of Zahid Hasan, a physicist at Princeton University in New Jersey. *“Emergent phenomena in topological physics are probably all around us even in a piece of rock”*. This underlines how topological physics helps to gain a deeper understanding of the nature of matter.

Topological physics is truly exploding: it seems increasingly rare to see a paper on solid-state physics that does not have the word topology in the title [51]. The expression “topology” seems to be all the rage at the moment, but it is often used improperly and to excess because there is some confusion between topology and geometry. We discuss concepts which are to be understood at an intuitive level (more precise definitions, theorems and proofs are required). From mathematics, there are three notions of equivalence between metric spaces which are of increasing “importance”: Homeomorphism concerns the topology → bi-Lipschitz transformation which is familiar to physicists as “fractal world” → isometries (e.g., crystallography for physicists) (see the Appendix A for the formal definition). Topology is concerned with those properties of geometric figures that are invariant under continuous transformations (homeomorphism). In other words, topology is the study of the geometrical properties of an object that remains unchanged when continuously transforming the object [52]. Intrinsic and extrinsic geometry are definitively associated to a metric space: a set together with a metric on the set. The metric is a function that defines a concept of distance between any two members of the set, which are usually called points (atoms in our case). Even though there is no frontier between topology and geometry in mathematics, this is not the case in physics. Geometry concerns the study of the lattice defined by a set of points with known coordinates, while topology targets the thermodynamics and especially the kinetics of the reaction/transformation of the material under external field (photon, temperature, particle irradiation, etc.). The driving force is that the time evolution follows some of the rules dictated by topology and especially the classification theorem [53]. As long as *GLC* appears during the transformation of an amorphous carbon under external field, it is a key issue to understand the topology rules for following this transformation, in particular, for evidence of the graphitisation process (or not).

### 4.2. Dimension

The concept of dimension has many aspects and meanings within mathematics, and there are several very different definitions of what the dimension of a set should be. The intuitive feeling of dimension for physicists is called the topological dimension $d_{t}$. In carbon films, we have to consider $d_{t}=2$ (the graphene which is a flat surface embedded in $E^{2}$ Euclidean space) and $d_{t}=3$, when the curvature is non-zero such as fullerenes.

### 4.3. Connection between Thermodynamics and Topology

Except the phase transition that (often) corresponds to an energy jump, the evolution of the surface under an applied force is continuously deformed according to the principle of minimum energy path. In carbon, the continuous deformed lattice is associated to a change in the hybridisation according’s to *POAV* (Pi-orbital vector analysis) theory [54]. Then, the transformation (graphitisation or not) mimics a homeomorphism in the mathematics point of view. Note that not all the operations in topology have a physical meaning. *Two surfaces in space are homeomorphic if we can bend, stretch, squeeze or shrink one into the other and/or if we can cut one and then, after some bending, stretching, squeezing or shrinking, glue it back together (making sure to join the points on either side of the cut exactly as beforehand) to form the other [55]*. If we cut some bonds, it costs a lot energy even though the gluing restores the energy (the gluing operation is illustrated below). The net balance energy is zero but the barrier is large. We restrict to “equivalent shapes” where if and only if one may be continuously deformed into the other without any cuts, self-intersections or singular points in the objects. In other words, we just consider in physics the homotopic transformation. Two continuous paths in a topological space are homotopic if one path can be continuously transformed to the other (in other words, cuts are avoided because of the excessive energy cost to do them).

### 4.4. Topology of Surfaces: Geometry Aspect

We recall the background of the topology dedicated to surfaces. Readers can find further information in specialised books [56,57,58].

#### 4.4.1. Curvature

Two important measures of curvature are under consideration. The mean curvature*H* is defined to be the average of the principal curvatures $k_{1}$ and $k_{2}$

(1) $$H=\frac{1}{2}\left(k_{1}+k_{2}\right)$$

The Gaussian curvature *K* is defined to be their product

(2) $$K=k_{1}k_{2}$$

The Gaussian curvature for example determines whether a surface is locally convex (*K* > 0) or locally a saddle (*K*< 0). The area A=∫dA, where *dA* is the area element, is preserved by isometries. The total Gaussian curvature G=∫KdA is topological invariant for a closed surface (see the Gauss–Bonnet theorem). The total mean curvature H=∫HdA depends on the external geometry of the surface. The Gauss–Bonnet theorem is a relationship between surfaces in differential geometry. It is a bridge between geometry (surface curvature) and topology (Euler characteristic, see below). The most important notion of curvature for us is the Gaussian curvature which measures the deviation of formulas for triangles from the Euclidean ones. It allows us to relate the differential geometry of the surface to its topology. For compact surfaces *S* (no boundary, see below), the Gauss–Bonnet theorem states that, by integrating the Gaussian curvature *K(s)* over an orientable surface,

(3) $$\int_{S}dsK\left(s\right)=2\pi \chi \left(S\right)$$

χ(S) is the Euler characteristic for the surface *S*. For a sphere, integration yields 4π and 0 for the plane and the torus and −4π for the double torus. The mean curvature of a surface at a point is an extrinsic quantity. The Gaussian curvature is an intrinsic quantity. The principal curvatures are extrinsic quantities.

#### 4.4.2. Euler Characteristic

The Euler characteristic is a topological invariant and also a homotopy invariant. Euler’s formula states for a polyhedron with a collection of vertices *V* edges *E* and faces *F*

(4) V−E+F=2

We also have the conditions: an edge must start and finish to an edge; when two edge meet, they must meet in a vertex; and faces must be distorted polygons. The connection with a surface is obvious; if we remove one face, the remaining polyhedron can be flattened into the plane. The general form for Euler’s formula is written as

(5) $$\sum_{k}F_{k}-E+\sum_{n}V_{n}=\chi$$

where *k* corresponds to *k*-membered rings (*k* = 4–8 in standard $sp^{2}$ carbon compounds with *k* = 6 in graphene) and *n* is the coordination number (*n* = 3 in $sp^{2}$ carbon).

The Euler characteristic is related to the genus *g* (the number of torii in a connected sum decomposition of the surface; in other words, the number of “holes” or “handles”) by the simple formula for a closed orientable surface

(6) χ=2(1−g)

where *g* is the genus. For a closed non-orientable surface (*k* is the non-orientable genus),

(7) χ=2−k

Table 2 gives some topological invariants for common surfaces.

The Euler characteristic is well defined for carbon clusters having a positive curvature (fullerenes, nanotubes, onions, etc.) or *TPMS*. As long as *GLC* is a complex lattice, the Euler characteristic becomes a “local” parameter. One defines χ in algebraic geometry (in terms of Betti numbers) where, assuming a finite number of singularities [59,60] (known as the “mountain dweller relation”),

(8) χ=v−s+t

with *v*,*t* and *s* the valleys, tops and saddles, respectively. Table 3 gives *v*, *t* and *s* for the four square gluing diagram (see Section 4.6).

This procedure is more appropriate for complex structures such as *GLC* where the geometry and the topology are defined “slab” by “slab”. Then, in *GLC*, the topological invariants are defined locally into a slab.

#### 4.4.3. Geometry of *2D* Surface

In mathematics, and more precisely in geometry, Poincaré’s uniformisation theorem asserts that any surface admits a Riemannian metric of constant curvature. Geometric classification of surfaces are:

(i) the sphere of Gaussian curvature +1;
(ii) the Euclidean plane of Gaussian curvature 0; and
(iii) the hyperbolic plane of Gaussian curvature −1.

In *2D*, all Riemannian surfaces are homeomorphic to the three constant curvature geometries $S^{2}$, $E^{2}$ and $H^{2}$. The two former are embedded in $E^{3}$ and can be easily represented in Euclidean space. $H^{2}$ is not embedded in $E^{3}$ and the polynomial equation is a sufficient approximation for physicists (who like approximations). All the $sp^{2}$ forms (each atom has a three-fold coordination) are described as a polygonal tiling of the surface, where each vertex corresponds to a carbon atom, each edge to a covalent bond and each polygon to a carbon ring. The surface covered by the polygonal tiling of carbon rings is characterised by its connectivity or order of connection *n*. *GLC* is definitively a *2D* surface with different local properties characterised by the local or global Gaussian curvature.

### 4.5. The Classification Theorem

Every proof of the classification theorem [58] for compact surfaces comprises two steps [53]: (1) The topological step consists in showing that every compact surface can be triangulated. (2) The combinatorial step consists in showing that every triangulated surface can be converted to a normal form in a finite number of steps, using some (finite) set of transformations.

**Theorem** **1** ([61,62]). *Every compact (connected) surface is equivalent to one of the following three types of surfaces (see below for the definition):*

*(i)*  *a sphere;*
*(ii)*  *a connected sum of projective planes (if it is non-orientable); or*
*(iii)*  *a connected sum of torii (if it is orientable and not a sphere).*

*A compact surface is classified in terms of its boundary number β, its orientability number ω and its Euler characteristic χ*. These numbers are topological invariants and are preserved under homeomorphism. Then, we can speculate that, under laser annealing, these numbers remain constant during a “continuous deformation” (called homotopy). This is of prime importance for a comprehensive study of the non-graphitisable versus graphitisable process.

For the following, we need other definitions:

For two surfaces *A* and *B*, the connected sum of *A* and *B* is formed by cutting a disk from *A* and a disk from *B* and gluing the surfaces together along the boundary.

One obtains a surface with a boundary by taking any surface without a boundary and punching some holes in it by removing open discs. Using connecting sum, two surfaces can be connected by “gluing” the edges of the open disks together.

### 4.6. Planar Model

**Figure 6:**
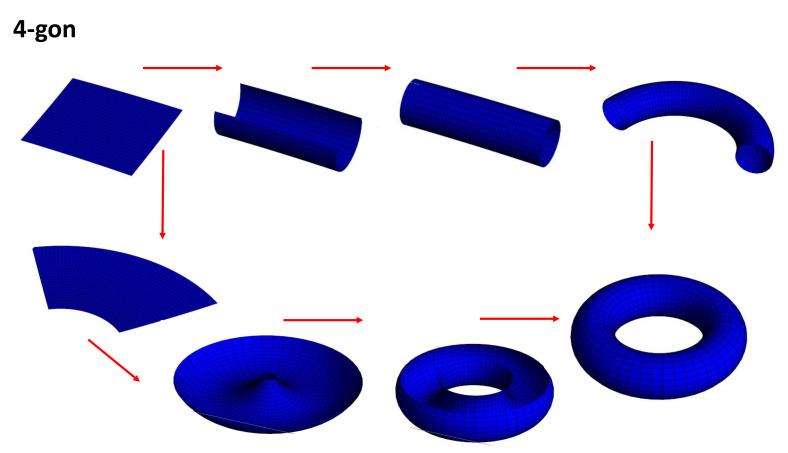
Two pathways for the flat torus formation corresponding to *BVK* conditions or Wallpaper group in crystallography.

A planar model of a surface is a polygon whose vertices and edges are identified, or glued together,“ in some specified way” (cut and paste methods are used in the proof of the classification of surfaces). This abstract concept is the basis of the common crystallography where polygons are duplicated with some rules. We can create a cylinder by using a piece of paper and gluing the ends together. Stretching the cylinder and gluing them, the two opposite sides gives a flat torus (a donut from a cooking point of view). Native people of the 2-torus look forward and see them from behind. They have the illusion of seeing a copy of them. The flat torus mimics the periodic BVK (Born–Von Karman) conditions in solid state physics commonly observed in standard crystallography (A *2D* crystal is a periodic arrangement of units, e.g., squares). The torus is homeomorphic to the unit square [0,1]×[0,1] with opposite sides identified. Following the homeomorphism transformation rules, different pathways are possible given at the end the flat torus (see Figure 6).

**Figure 7:**
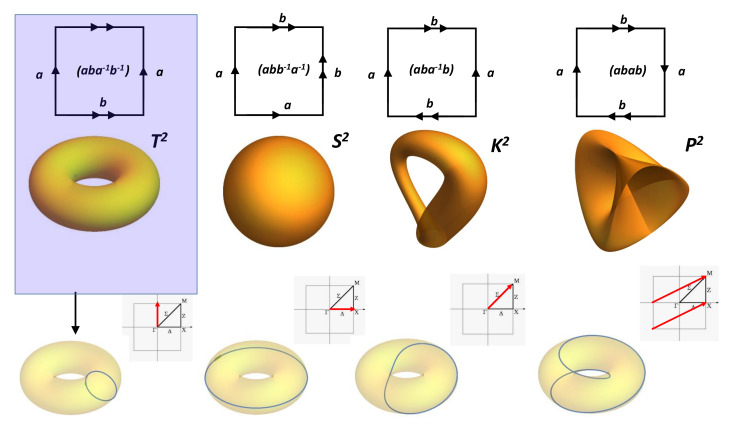
Four cases for the square gluing. The gluing instructions are given by arrows. A gluing diagram for a polygon is an assignment of a letter and an arrow to each edge of the polygon. The shaded figure (top left) gives the *BVK* conditions in standard crystallography. The electronic wave functions trajectories in the momentum space that correspond to special geodesics are drawn on the torus. The direction in momentum space is given in the Brillouin zone for selected commensurable paths.

**Figure 8:**
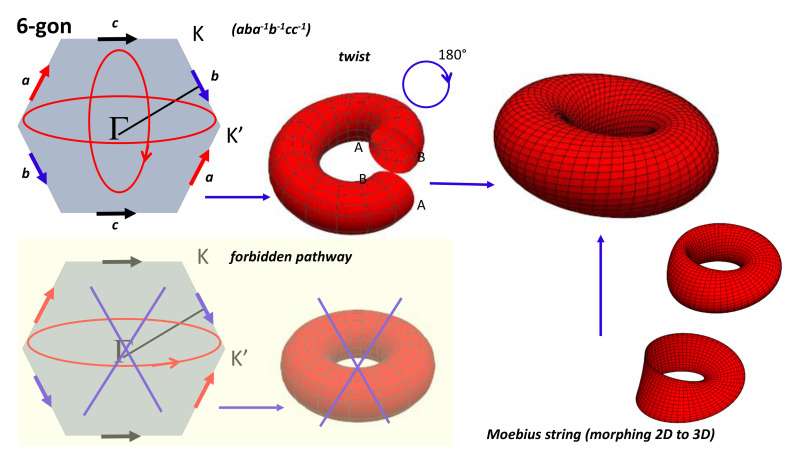
Gluing instructions $\left(aba^{-1}b^{-1}cc^{-1}\right)$ for a hexagon corresponding to the hexagonal tiling in $E^{2}$, the prototype of graphene (wallpaper group). (Bottom left) Gluing instructions are not compatible with a simple flat torus. Gluing “c” gives a cylinder. Direct paste of the cylinder edges are not compatible with gluing instructions for “a” and “b”. After a twist of $180^{\circ }$, gluing instructions are compatible given a twisted torus. The twisted torus can be seen as morphing a Möbius Strip into a torus (O. Seipel Wolfram Demonstrations Project Open content licensed under CC BY-NC-SA). Note that the fundamental group basis $(aba^{-1}b^{-1}cc^{-1})\ldots$ is canonical since two loops intersect only at the base point [66].

Other conditions than *BVK* are possible from a mathematical point of view. This is the so-called problem of the gluing diagram. Even though it is difficult to build up a crystal with other periodicities, let us keep in mind that the inhabitant in the flat torus pathway is an electron’s wave function moving in momentum space. In certain conditions (large spin–orbit coupling in *3D* topological insulator, band inversion and heavier compounds [63]), electronic wave functions “see” other gluing instructions. Other possibilities for the square gluing can be considered [64,65]. There are six ways for gluing a square. They give four classes: Klein bottle, $RP^{2}$ (projective surface), sphere and torus (Figure 7). Figure 7 shows the four ways for gluing a square to make a closed surface. Both torus and sphere are embedded in $E^{3}$ Euclidean space, while Klein bottles are not (a Klein bottle is embedded in $E^{4}$). For example, the Klein bottle (pseudo)representation in $E^{3}$ needs to allow the surface to intersect itself. A projective plane is a two-dimensional projective space, but not all projective planes can be embedded in three-dimensional projective spaces. Note that a Klein bottle has no boundary as observed in a sphere, which is an orientable surface. Other surfaces can be obtained starting with polygons (with even number of sides) and pairwise gluing together the edges of the polygons. In graphene, the topology corresponds to the case of hexagons. The result is a “twist” torus which is similar to morphing a Möbius strip into a torus (the original *2D* surface is just “blown up” to *3D* without considering homeomorphism) (Figure 8).

**Figure 9:**
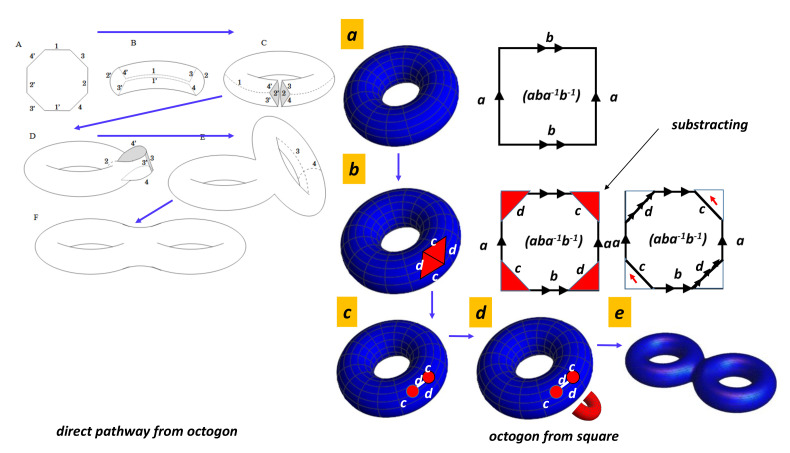
Two pathways for gluing an octagon: (**Left**) direct pathway [67]; and (**Right**) pedestrian pathway starting with a square. The octagon comes from a square where fours corners at cut off (red regions). Gluing each pair of sides give the flat torus minus a rhombohedron corresponding to the four corners gluing. After gluing *D* sides, one obtains two new gaps whose boundaries correspond to C sides. C sides are then glued together giving a second torus as shown in the figure. One obtains by homeomorphism and connected sum a double torus with a genus *g* = 2.

If we consider the periodic arrangement of atoms, the single way is the torus, but, if we consider the properties of the electronic wave functions (see, e.g., the case of topological insulators), other ways can be considered. Figure 8 shows the case of the hexagon (which is the template for graphene). The case of the pentagon is interesting. One obtains a punctured torus: the pentagon is the homeomorphic of the hexagon where we “subtract” a triangle. Gluing the triangle gives a cone. Because of the “subtracted” cone, this is not a closed surface (this is a surface with a boundary). Another interesting case is the octagon tiling (in hyperbolic space, Figure 9). We restrict now to the space resulting from gluing opposite edges of an octagon assuming “antipodal edges” as when building a torus from a square. The result is indeed a two-holed torus. In a similar way, one could obtain a three-fold torus by identifying the twelve edges of a dodecagon in pairs, as well as, in general, an n-fold torus from a 4n-sided polygon, for all natural numbers n. As long as even parity is needed for periodic conditions, joining the opposite edges of odd parity polygons such as pentagons is not possible. One solves the problem by introduction an edge which is a forbidden region in the momentum space. One obtains a punctured torus.

### 4.7. Special Points

**Figure 10:**
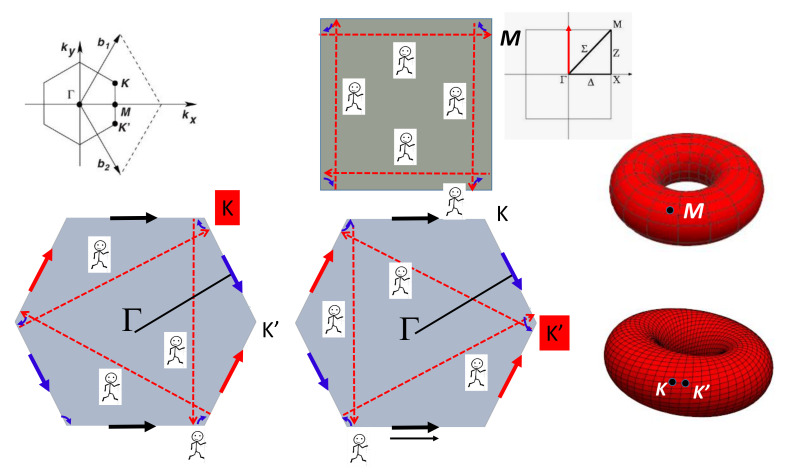
Comparison between the square (upper side) and the hexagon (lower side). In the square, the inhabitant occupies the four corners at the “same time” since all M points are equivalent (inhabitant visits the four corners). In the hexagon, one needs two “entangled” inhabitants for spanning the six corners in the hexagon (each inhabitant visits three corners). The “trajectories” are symmetric. We need two distinct points K and K’ at the origin of the geodesics.

Coming back to the square. Imagine an inhabitant living at *M* point in the Brillouin zone. First, check that the inhabitant is the same. To do this, he can move in momentum space with the periodic conditions. Starting from one selected *M* point, the inhabitant visits all four corners labelled *M* before coming back home. The inhabitant position in the torus is given arbitrarily in Figure 10 (that depends on the initial conditions). If we consider an inhabitant in the hexagon, the problem is different. An inhabitant living at *K* point visits three sites before coming back home. Likewise, an inhabitant living at *K’* visits the three other points in the hexagon. From a topological point of view, one needs two inhabitants for visiting the six corners. These two inhabitants are entangled but different (twin state). This is the keypoint for the Dirac points at *K* and *K’* [68]. The graphene properties at the special *K* and *K’* points are the topological properties of the hexagon. The figure shows the two inhabitants in the torus which are entangled but a different position in the torus (they are symmetric). This is the starting point for topological aspects of *2D* graphene-like materials [69].

### 4.8. Periodicity: Space Tiling

**Figure 11:**
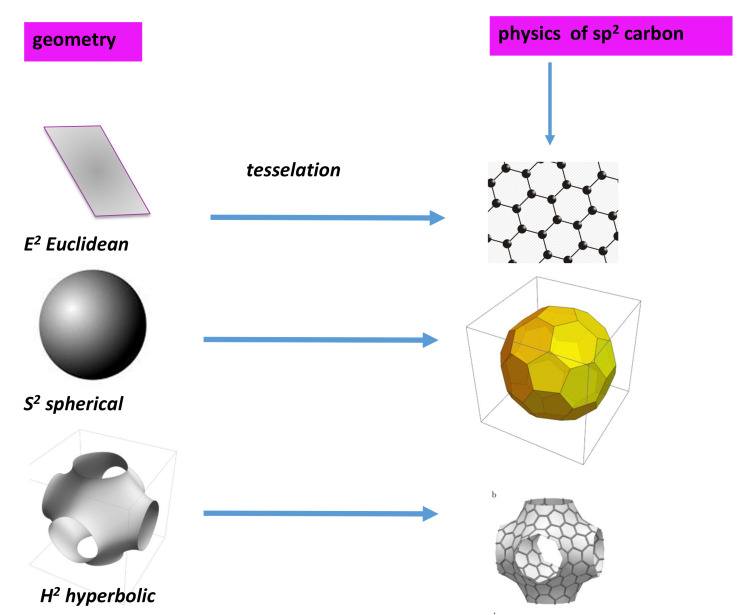
From continuous to discrete geometry in the three spaces: graphene (hexagon tiling), $C_{60}$ (hexagon and pentagon tiling) and *TPMS* (hexagon and n-gon n>7 tiling).

In Section 4.4.3, the geometry of surfaces deals with a “continuous or smooth surface”. In *GLC* and other compounds, the geometry deals with a discrete surface. We need a tiling or tessellation of a flat surface, which is the covering of a plane using one or more geometric shapes, called tiles, with no overlaps and no gaps (Figure 11). Prior to discussing the periodicity, one can introduce some general tools (proofs are beyond the scope of this paper and can be found in seminal geometry books).

#### 4.8.1. The Local Gauss–Bonnet Theorem

The Gauss–Bonnet theorem (i.e., the global Gauss–Bonnet theorem) is introduced in Equation (3) for smooth surfaces. For discrete surfaces (tiling), we use the local Euler characteristic associated to the symmetry operator (translation, rotation, inversion, etc.). The local Gauss–Bonnet theorem relates the curvature integrated over the surface area within a surface patch *P* bounded by a p-sided polygon with geodesic edges (the geodesic curvature of a curve is a measure of the amount of deviance of the curve from the shortest arc between two points on a surface) and internal vertex angles $v_{i}$: [70,71].

(9) $$\chi_{loc}=\left(2-p\right)\pi +\sum_{i=1}^{p}v_{i}$$

Conway introduced the orbifold (i.e., the orbit manifold introduced by Thurston) notation [72,73] for representing types of symmetry groups in two-dimensional spaces of constant curvature. Groups representable in this notation include the point groups on the sphere $S^{2}$, the frieze groups (surface repetitive in one direction) and wallpaper groups of the Euclidean plane $E^{2}$ and their analogues on the hyperbolic plane $H^{2}$. The orbifold first introduced by Thurston [72] is obtained by taking the quotient of the Euclidean space by the group under consideration ($E^{2}$, $S^{2}$ and $H^{2}$). Classification of two-dimensional crystalline patterns using orbifolds is given in the seminal paper by Hyde et al. [70]. Conway et al. [73] calculated the contributions to the total Euler characteristic $\chi_{loc}$ of an orbifold due to all possible orbifold features (Table 4).

An integer *n* to the left of an asterisk indicates a rotation of order *n* around a gyration point. An integer *n* to the right of an asterisk indicates a transformation of order *2n* which rotates around a kaleidoscopic point and reflects through a line (or plane). Note that, for a sphere, $\chi_{loc}^{i}=\chi =2$ (g = 0). One defines the fractional Euler characteristic $\chi_{o}$ summing all the Euler characteristic $\chi_{loc}^{i}$ (see Section 5.7.2)

(10) $$\chi_{o}=2-\sum_{orbifoldi}\chi_{loc}^{i}$$

#### 4.8.2. *2D* Crystallography

In classical crystallography (that means in Euclidean space), the curvature is zero and the *2D* crystal symmetry is given by the so-called wallpaper group. In *2D*, all Riemannian surfaces are homeomorphic to the three constant curvature geometries $E^{2}$, $S^{2}$ and $H^{2}$. We summarise some plane, elliptic and hyperbolic groups in Table 5, Table 6 and Table 7, respectively, for the Coxeter class.

**Figure 12:**
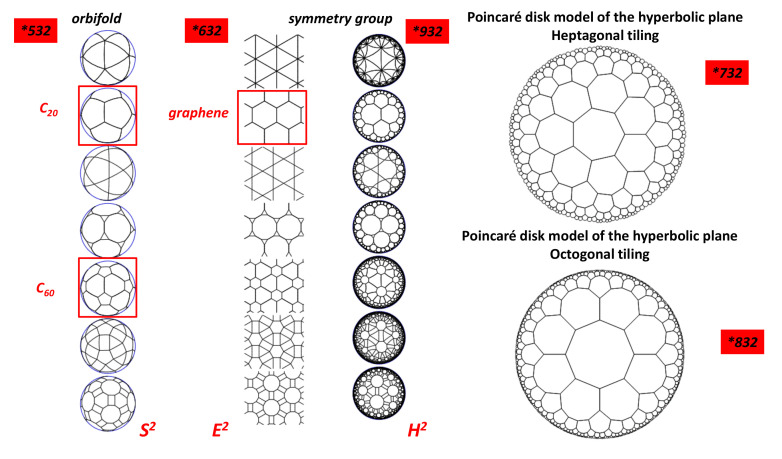
(**left**) Different orbifold (symmetry group, $532^{*}$, $632^{*}$ and $932^{*}$) corresponding to $E^{2}$, $S^{2}$ and $H^{2}$, respectively. The two fullerenes $C_{20}$ ($I_{h}$) and $C_{60}$ ($I_{h}$) belong to the $532^{*}$ symmetry group. Graphene belongs to the $632^{*}$ symmetry group [76]. (**right**) Poincaré disk model of the hyperbolic plane showing the tiling with heptagons or octagons (Platonic tessellation). The case of octagon tiling corresponds to the double torus in Figure 9.

Figure 12 shows selected configurations in the three spaces. We focus now on the $H^{2}$ group. The octagon gluing depicted in Section 4.6 corresponds to the octagonal tiling, which is a regular tiling of the hyperbolic plane. This is the simple way to observe that genus is at least two in hyperbolic space (octagon gluing gets a double torus). It is represented by a Schläfli symbol of 8,3, having three regular octagons around each vertex. The case of *TPMS* tiling the hyperbolic space $H^{2}$ is discussed in [75]. *TPMS* have a genus g≥3. The observed cavities in $R^{3}$ “created” by hyperbolic space characterise *GLC*. In other words, n-gons with n≥7 are necessary to observe low density carbon compounds with cavities.

### 4.9. *TPMS*

**Figure 13:**
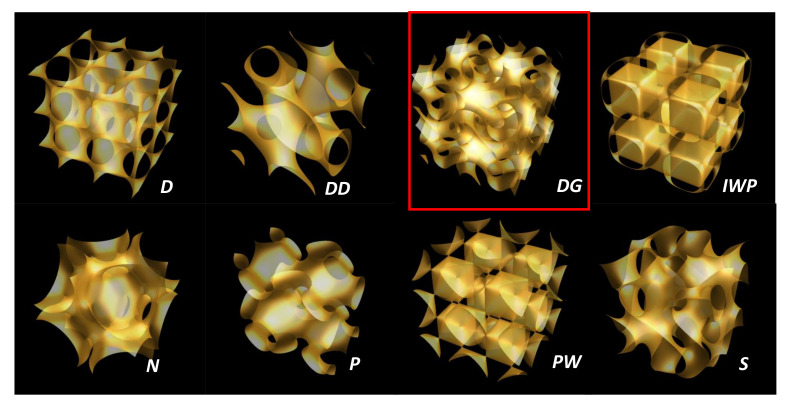
Some *TPMS* embedded in $E^{3}$. We must keep at mind that this is a pseudo-representation (Hilbert’s theorem states that $H^{2}$ is not embedded in $E^{3}$). In the following, we focus on the Schoen gyroid surface labelled “DG” (see the frame).

*TPMS* [77,78,79,80] have two properties: translational symmetry in three axes and a minimal surface, which is a surface that locally minimises its area. This is equivalent to having zero mean curvature. *TPMS* are ubiquitous in many fields. For example, the areas of zero electrostatic potential within an array of electric charges in an ionic crystal can be represented as a zero equipotential surface (*ZEPS*), which separates space into domains of positive and negative potential. These areas coincide with *TPMS* [81]. *TPMS* are oriented surfaces in $E^{3}$ that have constant vanishing mean curvature *H* = 0 and that are periodic with three linearly independent lattice vectors. Gaussian curvature is negative, which corresponds to hyperbolic space. The minimal genus of triply periodic minimal surfaces is 3 (to ensure triple periodicity). Any *TPMS* divides $E^{3}$ into two domains *K* and $\bar{K}$ in positive and negative normal direction from S, respectively. Both domains are continuous (i.e., connected), hence the term bicontinuous. In general, *K* and $\bar{K}$ may not be congruent [82]. Among the huge zoology in *TPMS* (see Figure 13), the gyroid separates space into two oppositely congruent labyrinths of passages. This structure is still interesting because of the chirality, and the gyroid structure is closely related to the $K_{4}$ crystal [83] (Laves phase, see Section 5.5). The gyroid has space group $I4_{1}32$ (No. 214). The Schoen gyroid (*G*) [77] may be approximated using the periodic nodal surface expansion. Integration of the Enneper–Weierstrass canonical representation of the *G* minimal surface in $R^{3}$ with the Weierstrass function enabled us to obtain analytical expressions for the Cartesian coordinates of the fundamental patch of the surface [84]. The fundamental equations are written as follows

(11) $$x=\exp\left(i\theta \right)ℜ\int_{\omega 0}^{\omega }\left(1-\tau^{2}\right)R\left(\tau \right)d\tau$$

(12) $$y=\exp\left(i\theta \right)ℜ\int_{\omega 0}^{\omega }i\left(1+\tau^{2}\right)R\left(\tau \right)d\tau$$

(13) $$z=\exp\left(i\theta \right)ℜ\int_{\omega 0}^{\omega }2\tau R\left(\tau \right)d\tau$$

with

(14) $$R\left(\tau \right)=\frac{1}{\sqrt{\tau^{8}-14\tau^{4}+1}}$$

The Bonnet angle θ is 0 and $38.0147^{\circ }$ in *D* surface and gyroid surface, respectively [84]. Other methods are under consideration [80]. A trigonometric approximation given by the lowest-order terms of a Fourier expansion of Schoen gyroid surface is written as [85]

(15) $$\cos\left(x\right)\sin\left(y\right)+\cos\left(y\right)\sin\left(z\right)+\cos\left(z\right)\sin\left(x\right)=t{|}_{t=0}$$

Crystallographic structures of $sp^{2}$ hybridised carbon need a tessellation of the minimal surfaces. As discussed above, we need k-gons with k≥7 at least. The number of hexagons, which is the standard tessellation of a flat surface, is free (see Equation (19) with *k* = 6). Euler’s relationship is written as follows (Equation (5)):

(16) $$\sum_{k}F_{k}-E+\sum_{n}V_{n}=\chi$$

with an additional relation

(17) $$E=\sum_{n}\frac{n}{2}V_{n}$$

The $sp^{2}$ hybridisation needs *n* = 3 (thus, *E* = 3/2V); each vertex shares three edges, and one edge connects two vertices. Moreover, in a convex polyhedron with *n* = 3, the vertex number *V* is related to the number of k-membered polygons by the relation

(18) $$3V=kF_{k}$$

For a tetrahedron, V=4, k=3 and $F_{k}=4$; for a cube, V=8, k=4 and $F_{k}=6$; for a dodecahedron, V=20, k=5 and $F_{k}=12$, etc. Combining Equations (16)–(18) gives [22]

(19) $$6\chi =\sum_{k}\left(6-k\right)F_{k}$$

**Figure 14:**
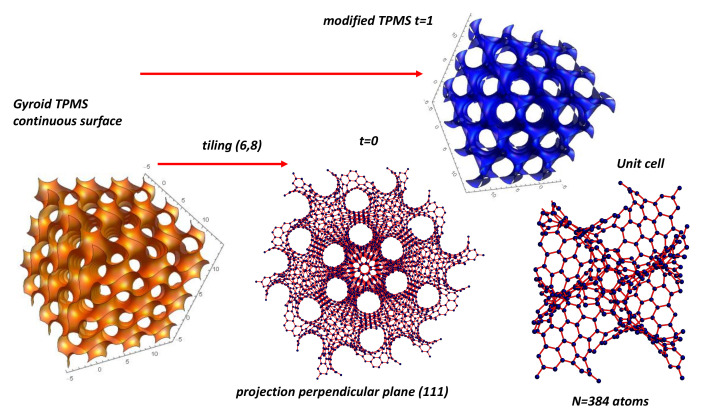
Gyroid *TPMS* with N = 384 atoms after tiling with hexagons and octagons (see Table 8, last line). The modified structure with *t* = 1 is discussed below.

**Figure 15:**
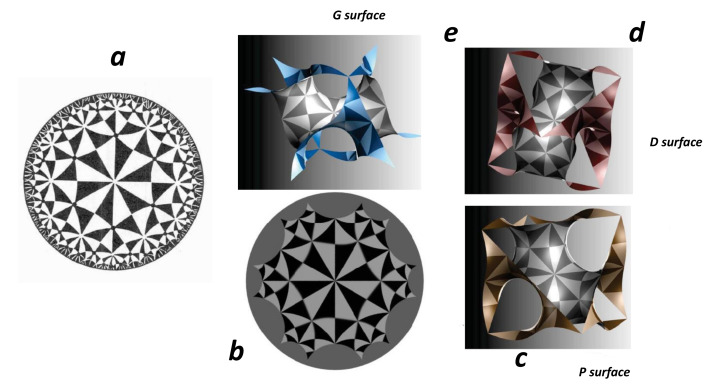
(**a**) Tiling of a hyperbolic plane with an orthoscheme triangle having angles π/2,π/4 and π/6 (*246) orbifold. (**b**) Region corresponding to the *P-D-GTPMS*. To do that, a part of the hyperbolic plane is cut out depending on the the type (*P*, *D* or *G*) [89]. (**c**–**e**) Projections in $E^{3}$ of *P*, *D* and *G TPMS*, respectively [90]. Figures (**c**–**e**) adapted from [91].

Since the genus is 3, the unit cell is a three-hole torus, and, according to Table 2, χ=−4. According to Euler’s theorem, the smallest Schwarzite is obtained with 24 heptagons ($k=7,F_{7}=24$) in the unit cell of both types (*D* or *P*), for any number of hexagons (except 1). This is one solution of the tessellation of the three-hole torus called the Klein ${7,3}_{8}$ tessellation [86] (*F* = 24, *V* = 56, *E* = 84). The table giving all platonic tessellations of genus 3 is presented in [86]. Among them, Equation (Section 4.9) gives an additional condition. This is the “opposite” case of the pentagonal dodecahedron (χ=2 and $F_{5}=12$) with 12 pentagons giving a positive curvature (and then a $sp^{3}$ hybridisation). *D*-type schwarzites have the structure of a diamond lattice so that the unit cell can be split into two identical elements having 12 heptagons each (two congruent labyrinths). P-type schwarzites have the structure of a simple cubic lattice. Strictly speaking, $sp^{2}$ hybridization needs *k* = 6 and *n* = 3. Other *k* membered rings bring strain energy and destabilize the lattice, the network stability needs *k* not too far from the primitive value *k* = 6. Platonic tessellation can be obtained with octagons (Dyck ${8,3}_{6}$ tessellation) [86]. The three smallest cases of Schwarzites and their crystallographic data are given in [87]. Table 8 displays the data for a gyroid structure with N = 384 atoms per unit cell using hexagonal and octagonal rings of carbon (Archimedean tiling $F_{8}=12$ see Figure 14 ). The number of Archimedean maps as a function of the genus in hyperbolic plane is given in [88]. Figure 15 displays the *D-P-G TPMS* with (*246) orbifold.

We note that *TPMS* are not surfaces of constant negative curvature in $E^{3}$ (just in $H^{2}$, but $H^{2}$ is not embedded in $E^{3}$) [92]. For *P*,*D* and *G* primitive *TPMS*, the variance [93]$\Delta =<{\left(K-<K>\right)}^{2}/<K{>}^{2}$ is minimal, its value is 0.219 and is the price to pay for a projection of a *TPMS* in $E^{3}$. Moreover, the crystallographic restriction of rotation orders to 2, 3, 4 and 6 ensures that many hyperbolic orbifolds cannot be embedded in $E^{3}$ to form three-periodic patterns.

Electronic properties of Schwarzite are discussed in the literature (for a review, see [87,94,95]). *color*Owens et al. [95] reported the electronic structure as a function of the distribution of non-hexagonal rings. All structures that have squares also have an occupied Dirac cone. Topological node-line semi-metal behaviour was evoked by Weng et al. [96], and similarly for the topology of the Dirac cone. According to the type of lattice, *TPMS* are found to be either metal-like or insulator-like.

## 5. *GLC* Properties: What Have We Learnt from Topology and Geometry

With this rudimentary knowledge of topology and geometry, we discuss now topological invariants deduced from the *uniformisation theorem* and the serious consequences in *GLC* properties.

### 5.1. Orientability Number

**Figure 16:**
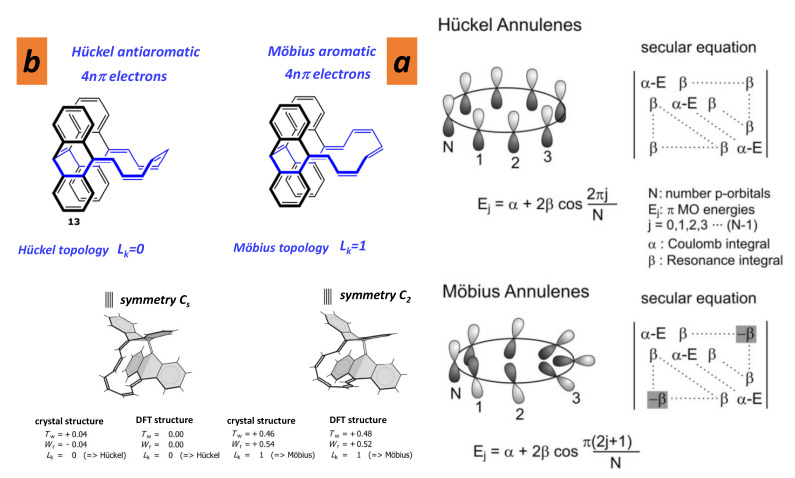
(**a**) Effect of the non-orientability in the Hückel scheme [97] showing the breakdown of the aromaticity rule. Note that there is a “−β term” (β is the overlapping integral) in the secular equation of the Möbius annulene, representing the sign inversion. (**b**) Topological parameters of the annulene derivatives with Möbius and Hückel topology. $L_{k}$, $T_{w}$ and $W_{r}$ come from knot theory. $L_{k}$ is the linking number for the parity giving the orientability number (*w* = 0 for even $L_{k}$ parity and *w* = 1 for odd $L_{k}$ parity), $T_{w}$ (twist) is a real number equal to the sum of the dihedral angles of the vectors normal to the π plane and $L_{k}=T_{w}+W_{r}$ [98,99]. (**a**) Reproduced with permission from Herges, Chem. Rev. [97]; copyright 2006, American Chemical Society. (**b**) Adapted from [99].

A surface is orientable if a person in the space cannot be moved continuously on that surface and back to their starting point so that they look like their own mirror image. Most of the common forms—fullerenes, nanotubes, onions, nanocones, graphene, etc.—are orientable. The most popular non-orientable form is the Möbius strip. Note that ribbons can be orientable when they form loops with an odd number of twists. Orientability number *w* is 0 if the surface is orientable and 1 if the surface is non-orientable. The orientability plays an important role in chemistry. For example, the annulenes with (4n+2)π electrons exhibit a more stable π system with Hückel “topology”, and those with 4nπ electrons prefer a Möbius twisted structure. Figure 16 displays the principle of Möbius topology [97,98]. A Möbius annulene will necessarily be larger than benzene in order to accommodate the ring twist while maintaining a reasonable steric overlap of the π-orbitals. There is no direct evidence of Möbius strips in *GLC* observed by *HRTEM*, but there is no proof that they are not allowed.

### 5.2. Boundary Number

**Figure 17:**
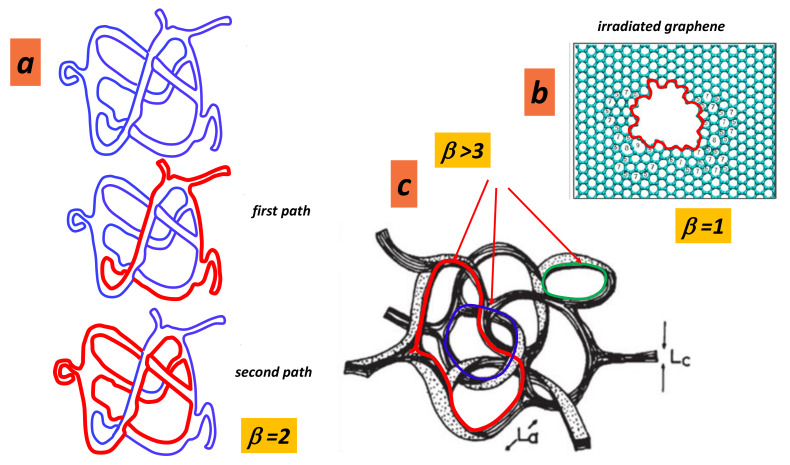
Some examples of boundary number: (**a**) β=2, the two paths are drawn; (**b**) β=1 in $Kr^{+}$ irradiated graphene after annealing [102]; and (**c**) some paths drawn in the Jenkins and Kawamura model [12] (the structure is too complex for a precise determination of β). (**b**) Reproduced with permission from Yoon, ACS nano [102]; copyright 2016, American Chemical Society.

**Figure 18:**
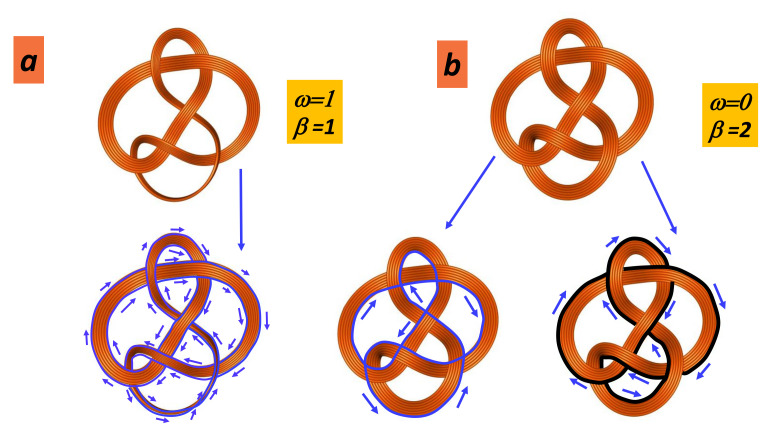
(**a**) Non orientable surface (Möbius string) showing one boundary (ω=1 and β=1). (**b**) Orientable surface showing two boundaries (ω=0 and β=2). The ribbon comes from “KnotPlot”, copyrighted by Rob Scharein.

Let us recall that a boundary is a line or border around the outside of a shape. It defines the space or area. A surface may or may not have a boundary. The number of these boundary components is the boundary number. Note that a boundary can be created by punching the surface with holes having a sizeable area (open disks). A surface with boundary is a topological space obtained by identifying edges and vertices of a set of triangles according to all the requirements of a surface except that certain edges may not be identified with another edge. These edges are called boundary edges and their vertices are called boundary vertices [100,101]. Confusingly, surfaces with a boundary are not surfaces from a mathematical point of view, but we continue to use the term “surface”. This operation costs a lot of energy (that corresponds in graphene to introducing a set of vacancies). At a medium range order, where *GLC* appears as a collection of spaghetti with ribbons tubes and other forms, this number takes some importance. Figure 17 shows some examples. Note that the boundary number depends on the orientability (see Figure 18).

### 5.3. Topological Invariants from Knot Theory: Graphitisation Process

**Figure 19:**
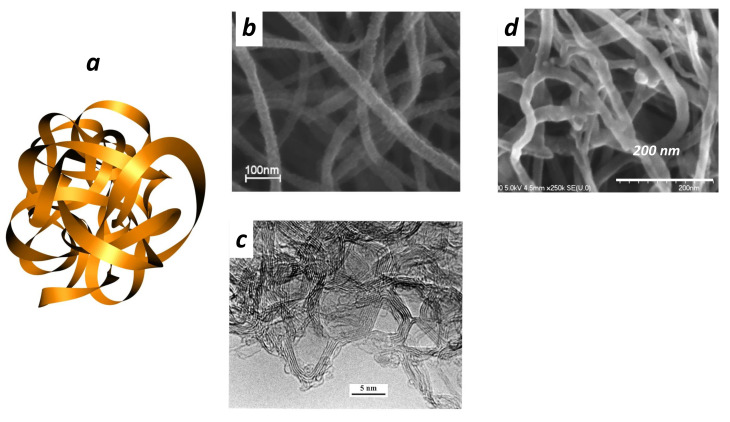
(**a**) Random knot [105] compared to several morphologies of carbon compounds; (**b**) nanotubes tangle [106]; (**c**) *GLC* [26]; and (**d**) multi-walled carbon nanotube buckypapers [107]. (**b**) Reproduced with permission from Li, Carbon [106]; copyright 2008, Elsevier, (**d**) Reproduced with permission from Smajda, Carbon [107]; copyright 2007, Elsevier.

**Figure 20:**
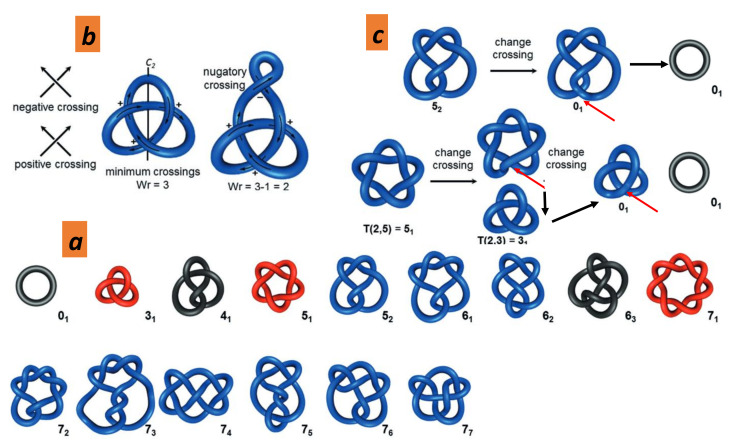
(**a**) Part of the Rolfsen knot table [108] giving the prime knot configuration up to seven crossing. (**b**) Conway’s fundamental tangles giving the sign (to do that, one defines a direction in the knot) illustration with a knot where additional tangle is introduced (that corresponds to type I Reidemeister move). The knot is the same, but the writhe number is different. The initial knot (the trefoil knot $3_{1}$) has a crossing number (all are positive) of 3 and writhe is 3. After additional tangle (noted nugatory crossing), the crossing number is 4 and the writhe is 3−1=2 since the new crossing is negative. (**c**) Transformation of a knot by changing the sign of the crossing (twice). The final result is a loop (the unknot $0_{1}$). Adapted from [104].

**Figure 21:**
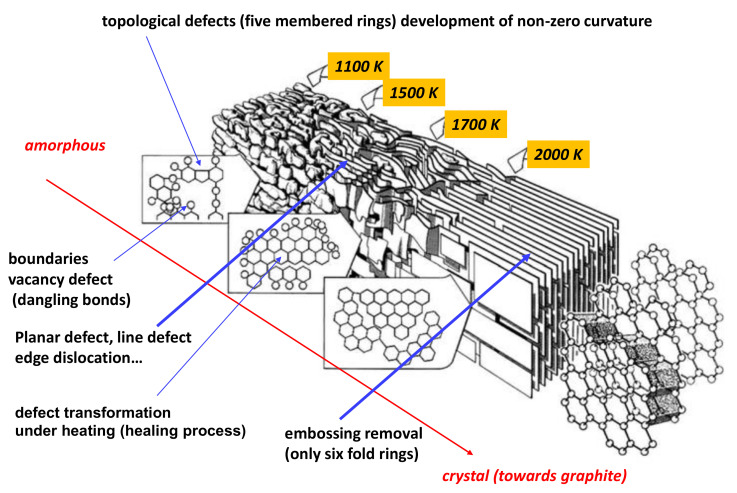
The Marsh–Griffith model of the carbonisation/graphitisation process [109]. The graphitisation needs a complete disentangle of the knots that underlines the role of the irreducible knots.

Figure 19 shows the knot formation in several carbon compounds. Under these conditions, it is logical to question the relevance of knot theory and its implication in the *GLC* property. Knot theory is beyond our discussion and we just consider some aspects. A theorem due to Horst Schubert states that every knot can be uniquely expressed as a connected sum of prime knots [103]. These irreducible knots are given by the Rolfsen knot table depicted in Figure 20 up to seven crossings. Of course, a knot can be untied if the loop is broken. The linking number is a numerical invariant that describes the linking of two closed curves in three-dimensional space. The linking number represents the number of times that each curve winds around the other. The linking number is always an integer, but it may be positive or negative depending on the orientation of the two curves. A twist knot is a knot obtained by repeatedly twisting a closed loop and then linking the ends together. The winding number is an integer representing the total number of times that the curve travels counterclockwise around the point. It is also topologically invariant and is an example of a topological quantum number in physics. Likewise, writhe is the total number of positive crossings minus the total number of negative crossings (see Figure 20a). In the Alexander–Briggs notation, links are written by the crossing number with a superscript to denote the number of components and a subscript to denote its order within the links with the same number of components and crossings. Note that the number of inequivalent prime knots dramatically increases with the crossing number (e.g., 3 with a crossing number of 6 and 165 with a crossing number of 10). Figure 20b,c shows that the determination of the prime knot (or connected sum) is a hard task [104]. In Figure 20b, the additional twisting in the loop (called nugatory crossing) changes the crossing number, but the knot remains the same. Similarly, in Figure 20c, changing the crossing sign induces a transformation towards another knot [104]. In summary, we can say with some confidence that the graphitic sheets or ribbons tangle depicted in Figure 19 give irreducible knots. These knots avoid the graphitisation process since graphitisation needs a complete disentanglement prior to a good stacking of graphene sheets under annealing (see Figure 21).

### 5.4. Electron Conductivity

**Figure 22:**
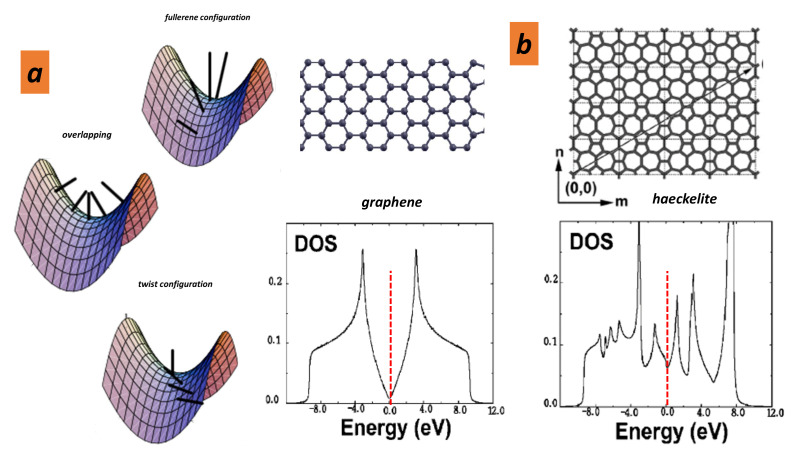
(**a**) π orbital misalignment in the crossing region as a function of the local curvature (positive or negative). The positive ones correspond to the case of fullerene family. π orbital is mapped by a line. Note the strong overlapping between π orbitals in the region of negative curvature. (**b**) Metal-like character observed in e-DOS in 5–7 haekelite [110]. Haekelite is a surface where two hexagons are replaced by a heptagon/pentagon pair. Note that 4–8 haekelite is an Archimedean tessellation, while 5–7 haekelite is not. Semi-regular tessellations (or Archimedean tessellations) have two properties: they are formed by two or more types of regular polygon, each with the same side length. Each vertex has the same pattern of the polygons around it. (**b**) Reproduced with permission from Terrones, Phys. Rev. Lett. [110]; copyright 2000, American Physical Society.

In the samples corresponding to Figure 5, the conductivity ratio between *2D*/*3D* amorphous region and *GLC* after annealing is about 1/60, indicating a metal-like character in *GLC*. In all $sp^{2}$ forms, the conductivity is due to π electrons. The conductivity mechanism in *GLC* is complex. Consideration is given to the topology of the whole network, which is “amorphous”, and the particular overlap of the π bonding electrons. A long time ago, Saxena et al. [29] reported a conductivity that is the sum of a temperature-independent contribution due to diffuse boundary scattering and a hopping contribution following a standard Hopping–Mott conduction law in $T^{-1/4}$. Ferrer-Argemi et al. [30] found that electrons and phonons are being thermally activated and that the lattice disorder dominates the scattering of the carriers. The low electron activation energy (8–14 meV) questions the conductivity behaviour at room temperature. Since the disorder depends on the preparation, we can attend a large spread in conductivity. The local curvature, in particular at saddle points (crossing region), changes the electronic structure: in some cases, twisted π states across the Fermi level result in metallic properties (see Figure 22a). Likewise, it has been recognised that a pentagonal/heptagonal pair that “mimics” a graphene sheet (haeckelite) gets a metal-like character [110,111] (Figure 22b). Metal-like character is also observed in other haeckelites [112]. Let us remember that 5–7 haeckelite is not a Archimedean tessellation (regular tessellations of the plane by two or more convex regular polygon) contrary to 4–8 haeckelite which is Archimedean. Then, the final result of the 5–7 pair is either a distortion of the regular polygon or a local non-zero curvature. Note that 4–8 haeckelite opens a gap of 0.36 eV with respect to the graphene (0 eV) [113]. Since the positive curvature opens a large gap (1.67 eV in $C_{60}$ [113]), we can say with some degree of confidence that negative curvature is responsible to the gap filling in agreement with the π overlapping in Figure 22a.

### 5.5. Isotropy

Coming back to the pioneering work of Franklin [7], the conceptual model has all the assets that we need. The keypoint is the crossing between graphitic sheets giving the global isotropy.

Sunada [114] proposed a theorem dedicated to isotropy (in the sense of mathematics) and valid for carbon networks.

*The degree of a three-dimensional crystal lattice with the strong isotropic property is three or four. The one with degree four is the diamond lattice, while the one with degree three is the $K_{4}$ lattice. Hexagonal lattice is a unique two-dimensional crystal lattice with the strong isotropic property*.

**Figure 23:**
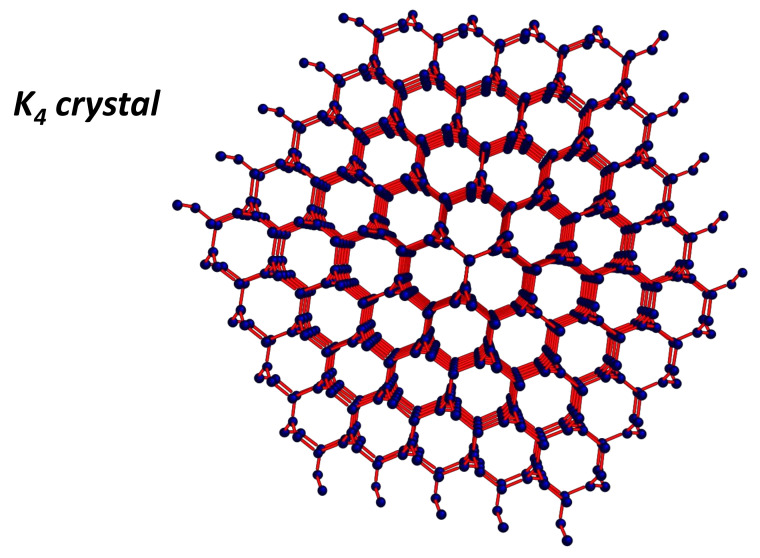
$K_{4}$ crystal is the prototype of an isotropic lattice (note that the “isotropy” in mathematics is stronger than the “common” isotropy in physics) with a connectivity 3 [115]. The structure is formed by a set of decagonal rings with a large open porosity and a very low density.

**Figure 24:**
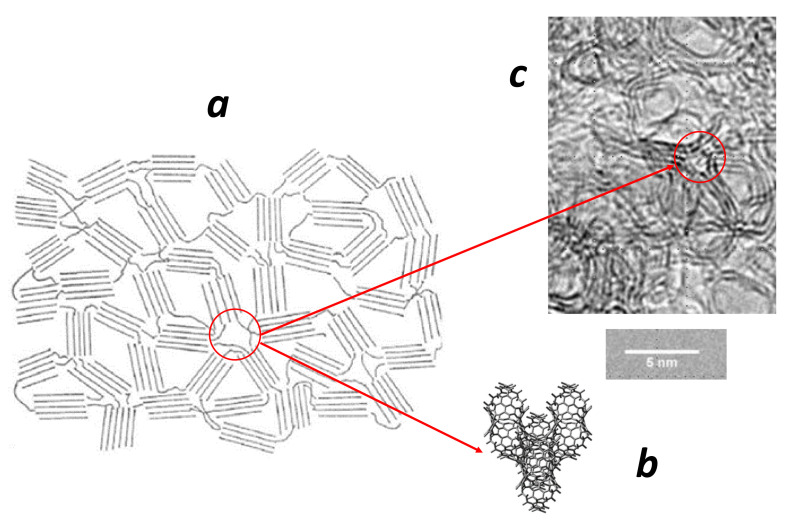
(**a**) Franklin’s view of the *GLC*; (**b**) part of *HRTEM* pattern corresponding to the *GLC* produced by *PLD* and laser annealing (see Figure 5); and (**c**) an elemental cell of a Schwarzite *TPMS* structure [22].

Degrees 4 and 3 correspond to $sp^{3}$ and $sp^{2}$ hybridisation in carbon form. The $K_{4}$ crystal is a regular graph of degree 3 and is constituted by a set of decagonal rings (Figure 23) [115]. There is a big difference between the $K_{4}$ crystal and the diamond crystal: $K_{4}$ has a chirality, while diamond does not. $K_{4}$ crystal, a purely mathematical chiral object, was first proposed for carbon [116], boron [117] or phosphorus [118]. Such a lattice is interesting as long as it can explained by a metal-like behaviour. However, $K_{4}$ belongs to the Kagome family [119] and presents large open pores permeable to gases. In other words, the *GLC* carbon definitively does not have the $K_{4}$ structure. Another possibility is the connection of graphitic sheets, wires or ribbons by an elemental cell of Schwarzite structure (Figure 24); thus, *TPMS* (Schwarzites) represented another path to explore. The concept of isotropy is well known in the standard theory of linear elasticity. Isotropy is assumed when elastic constants are the same for all orientation angles and the Poisson coefficient (which reflects the transverse strain response to the applied uniaxial load) is higher than 0.2 [120]. For example, pyrolytic carbon is partially isotropic: the Poisson coefficient is about 0.4 [121], but elastic properties depend on orientation angle and question the nature of isotropy in such materials. Theoretical Schwarzites structures have a Poisson coefficient ranging from 0.2 to 0.35 [122]. There is a large spread in the values reported for *GLC* because of the strong complexity of the network. *GLC* has a relatively low Poisson coefficient (0.17) [123] but is assumed “isotropic” [124]. Manoharan et al. reported a size effect with an increasing significance of surface elastic properties at the nanometer length-scale [125]. Once again, the network complexity between the graphitic sheets (curved, twisted, tangled or not) and the crossing region with a local hyperbolic geometry questions the validity of the elastic model. Hyperbolicity plays an important role. Li [126] predicted that hyperbolic two-dimensional carbon materials have an in-plane negative Poisson’s ratio behaviour.

### 5.6. Porosity versus Gas Diffusion

Diffusion (or permeability) in carbon compounds depends to the geometry of pores. Kowalczyk et al. [127] reported a theoretical study of the hydrogen storage in various carbon latices including gyroid (TPMS), diamond nanoporous carbon materials and nanotubes. Among them, TPMS present the lowest value of hydrogen adsorption. Song et al. [128] reported a permeability to helium of about $10^{-9}$ m${}^{2}$/s for low-density isotropic pyrolytic carbon and $5\times 10^{-11}$ m${}^{2}$/s for high-density anisotropic pyrolytic carbon. As a comparison, a IG-110 nuclear grade carbon has a permeability of $10^{-5}$ m${}^{2}$/s. Smajda et al. reported for multi-walled carbon nanotube buckypapers a permeability ranging 3–$12\times 10^{-9}$ m${}^{2}$/s [107]. Such values are far from the expected permeability in *GLC* ranging 1–$10\times 10^{-13}$ m${}^{2}$/s.

**Figure 25:**
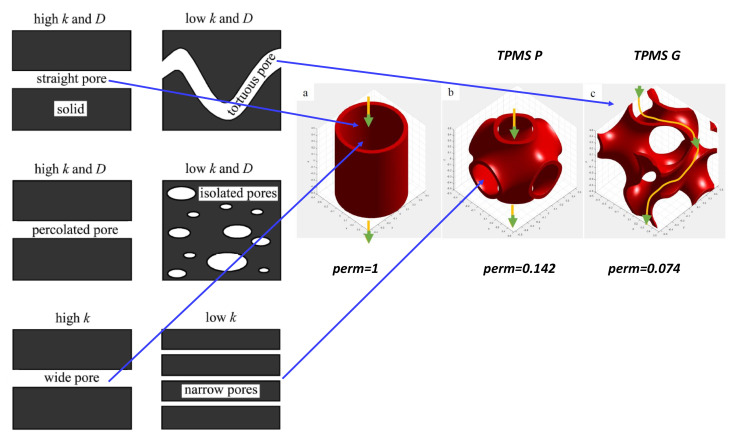
(**Left**) Synopsis of the effects of pore geometry on the macroscopic permeability (*k*) and pore fluid diffusivity (*D*) in carbon compounds [138]. (**Right**) The fluid permeability is illustrated for a cylinder and two *TPMS*. The permeability (k=perm) is less in *TPMS P* Schwarz structure because the open channel area is lower than the one in the cylinder. Gyroid *TPMS* has the lower permeability because of the large tortuosity. Reproduced with permission from Nakashima, Proceedings of the National Academy of Sciences [138]; copyright 2007, National Acad Sciences.

Porosity is probably the most amazing issue in *GLC*. *GLC* are totally impermeable to gas with a large open structure (see the low density and the *TEM* observation). The first naive answer is that *GLC* are impermeable to gas because of closed pores. This is in agreement with the apparent porosity of 0.2–0.4% in *GLC* as compared to 8–15% in impregnated carbon [129]. The permeability *K* is related to both parameters in the Tomadakis–Sotirchos model [130]: the fibre diameter $d_{f}$ (in *GLC*, it would be the diameter of the spaghetti) and the porosity ϵ [131].

(20) $$K=\frac{\epsilon }{8{\left(\log\epsilon \right)}^{2}}\frac{{\left(\epsilon -\epsilon_{p}\right)}^{\alpha +2}d_{f}^{2}}{{\left(1-\epsilon_{p}\right)}^{2}{\left(\left(\alpha +1\right)\epsilon -\epsilon_{p}\right)}^{2}}$$

where in *3D* random networks α=0.661 and $\epsilon_{p}=0.037$. As long as such models developed for fibre carbons can be applied in *GLC*, the low permeability suggests a porosity close to $\epsilon_{p}$. The issue is more complex and could be clarified thanks to the topology of mazes. One introduces an additional parameter: the tortuosity t. The tortuosity in gyroid TPMS is illustrated in Figure 25. For TPMS gyroid structures, diffusion properties can be controlled by a proper choice of gyroid size and density [132]. Furmaniak et al. [133] reported a theoretical study of Ar adsorption. These authors found a filling process depending on the pore diameter. Tortuosity factor and porosity are the two microstructure parameters that control the effective gas diffusion coefficient [134,135,136,137]. The Tomadakis–Sotirchos model expression for tortuosity is as follows

(21) $$t={\left(\frac{1-\epsilon_{p}}{\epsilon -\epsilon_{p}}\right)}^{\alpha }$$

**Figure 26:**
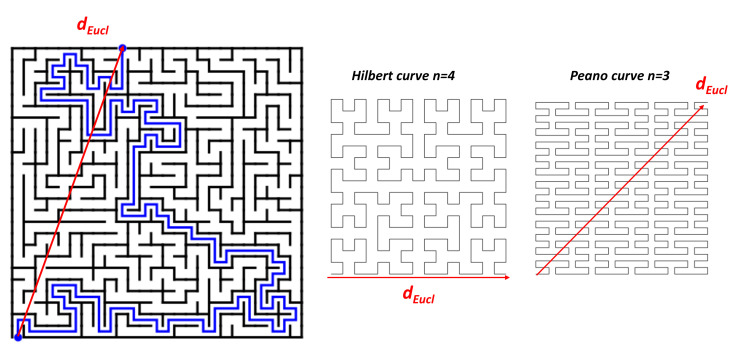
(**Left**) Tortuosity in a maze (t≃5.4). (**Right**) Tortuosity in two space filling (fractal) curves, Peano and Hilbert curves. The number of iterations is *n*.

Then, tortuosity tends to infinity (if $\epsilon ≃\epsilon_{p}$) in agreement with a low porosity. Figure 25 shows the effects of pore geometry on the macroscopic permeability (*k*) and pore fluid diffusivity (*D*) in the global permeability [138]. Unfortunately, the tortuosity t remains a poorly understood concept because the term targets different fields: geometrical, electrical, hydraulic, diffusional, etc. tortuosity. Ben Clennel [139] defined *tortuosity as the parameter which describes the sinuosity and the interconnectedness of the pore space as it affected transport processes*. From a topological point of view, tortuosity can be defined by the simple formula (geometrical aspect):

(22) $$t=\frac{\sum_{i}d_{i}}{d_{Euc}}$$

where *i* is the number of elemental steps. A *2D* example is given in Figure 26 where the tortuosity is just the percolating distance divided by the bird’s eye distance (the Euclidean distance). To illustrate this point, tortuosity can be easily computed in the special case of *2D* fractal space filling curves (Hausdorff dimension 2) [140] (Figure 26). For example, Peano and Hilbert curves gave tortuosity within a simple relationship ($d_{Euc}=1$ for the square side):

(23) $$t_{Hilbert}=\left(3+\sum_{i=1}^{n-1}2^{i}\right)/d_{Euc}$$

(24) $$t_{Peano}=\left(3^{n}+1\right)/d_{Euc}$$

where *n* is the iteration step in the fractal sequence: t increases rapidly with the iteration *n*

The increasing of the tortuosity dramatically affects the permeability because of the increasing of the net pathway and the fact that a rigid molecule can be stuck in a tight corner of the structure [137]. In addition, the gas–carbon interaction potential might be influenced by the surface curvature and ring structure, which could change the localisation of the electron density that affects the diffusion [141].

### 5.7. *GLC* Stability

#### 5.7.1. Willmore Energy

Willmore defined a functional on the space of embedding of a given surface. The Willmore energy $\bar{W}\left(S\right)$ is a quantitative measure of how much a given surface deviates from a round sphere. The Willmore conjecture can be viewed as a question about the “best possible” immersion of a surface in $R^{3}$ [142]. Willmore energy is written as

(25) $$\bar{W}\left(S\right)=\int_{S}H^{2}dA=\frac{1}{4}\int_{S}{\left(k_{1}+k_{2}\right)}^{2}dA$$

or another functional, umbillic or anisotropic energy,

(26) $$W\left(S\right)=\int_{S}\left(H^{2}-K\right)dA=\frac{1}{4}\int_{S}{\left(k_{1}-k_{2}\right)}^{2}dA$$

and the bending energy

(27) $$B\left(S\right)=\int_{S}\left(H^{2}-K\right)dA=\frac{1}{4}\int_{S}\left(k_{1}^{2}+k_{2}^{2}\right)dA$$

A remarkable property of Willmore energy is its invariance under conformal transformations of $R^{3}$ [143]. For a surface with mean constant curvature, using Gauss–Bonnet theorem in a closed surface,

(28) $$W\left(S\right)=\frac{1}{4}B\left(S\right)+\pi \left(2-2g\right)$$

**Figure 27:**
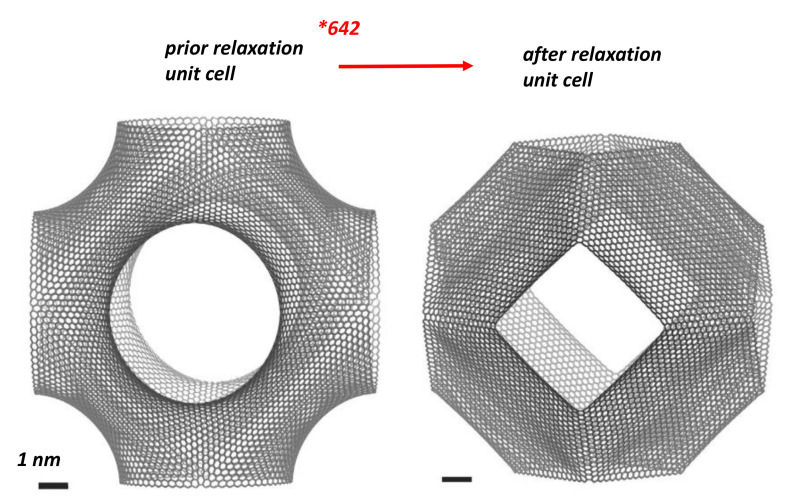
Relaxation of a unit cell (P-*TPMS*) showing the decrease of the Gauss curvature and the flatness after relaxation [122]. Both structures are topologically equivalent to the *P* surface. Reproduced with permission from Miller, Carbon [122]; copyright 2016, Elsevier.

It was conjectured by Willmore that $\bar{W}\left(S\right)\geq 4\pi$ for any compact surface in $R^{3}$ with the minima $\bar{W}\left(S\right)=4\pi$ in a round sphere. This minimal value explains why the equilibrium shape of the soap bubble is the sphere. Willmore conjectured (proved 2014) that for a torus $W\left(T^{2}\right)\geq 2\pi^{2}$ [144]. In carbon, one defines four asymptotic forms according to *K* value:

- The graphene with $k_{1}=k_{2}=0$ then K=H=0 and W(S)=0.
- The (spherical) fullerenes with $k_{1}=k_{2}=k$ everywhere, thus K>0 and H>0, $\bar{W}\left(S\right)=4\pi$ and W(S)=0.
- The nanotubes with $k_{1}>0$ and $k_{2}=0$, thus $W\left(S\right)=\bar{W}\left(S\right)$. The fourth belongs to the minimal surfaces, among them the triply periodic minimal surfaces (*TPMS*) with H=0, G<0 $\bar{W}\left(S\right)=0$. From a physical point of view *TPMS* are metastable structures. The excess in “strain” energy is evidenced after relaxation of a P-*TPMS* unit cell with boundaries [122] (Figure 27). Then, *GLC* cannot accommodate large holes that characterise giant Schwarzites (with a high number of hexagons) because of a complete relaxation. This is not the case for the smallest Schwarzites which are not smooth surfaces. Willmore energy states for smooth surfaces. One can define the discrete Willmore energy after tiling [145]. The physical stability is obtained minimising the discrete Willmore energy. We can discuss now the case of discrete surface in terms of local Euler characteristic.

#### 5.7.2. Defect Formula: “Mathematical” Stability

In order to classify the “stability” of a lattice (from a physical point of view), we can introduce the cost of a orbifold (that is interpreted with caution as a cost energy without formal proof). To do that, we use the defect formula [73] (Equation (10)):

(29) $$\chi_{o}=2-\sum \chi_{loc}^{i}$$

summed over all the characters of the orbifold symbol, where these defects (i.e., a local Euler characteristic) are tabulated in Table 4. $\chi_{0}$ is the orbifold Euler characteristic (or fractional Euler characteristic) and corresponds to the cost of the orbifold [146,147]. The trivial case is the sphere where χ=2, thus $\chi_{o}=0$. For example, the orbifold symbol of the graphene in the Conway notation is *632, the crystallographic symbol being *P6m*. The symmetry operators in graphene are, respectively, a reflection line (orbifold symbol *, $\chi_{loc}^{i}=1$) and three rotation centres with angles π/6 ($\chi_{loc}^{i}=5/12$), π/3 ($\chi_{loc}^{i}=2/6$) and π/2 ($\chi_{loc}^{i}=1/4$) (orbifold symbols 6, 3 and 2, respectively). Using Table 4, Equation (29) gives (see Table 7)

(30) $$\chi_{o}\left(graphene\right)=2+\left(-1-5/12-2/6-1/4\right)=0$$

Note that, for the Coxeter class, *2D* plane groups have a zero cost orbifold regardless of the type of tiling. Likewise, let us consider the fullerene $C_{60}$. We can project the net onto $S^{2}$, giving a symmetric pattern with identical vertices defined by a spherical triangular asymmetric domain, with 2, 3 and 5 mirror lines meeting at each vertex. The Coxeter orbifold is *235 or equivalently, the $I_{h}$ point group in classical crystallography in $E^{3}$. Then

(31) $$\chi_{0}\left(C_{60}\right)=2+\left(-1-1/4-1/3-2/5\right)=+1/60$$

In a physical description, $C_{60}$ is less “stable” than graphene. In *2D* spherical orbifolds, *235 has the low cost energy ($I_{h}$ symmetry), truncated octahedron formed by regular squares and hexagons (*m3m*) has a larger cost (*432 with $\chi_{o}=1/24$, see Table 6). Fullerenes with icosahedral symmetry are expected to be more stable than standard crystallographic forms.

**Figure 28:**
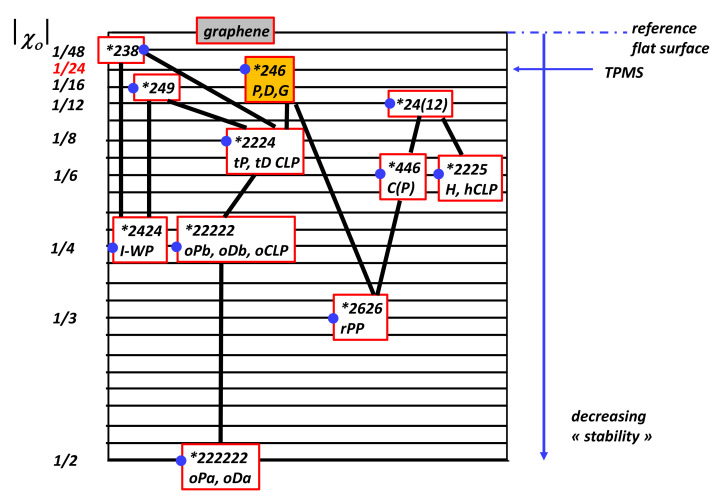
Group and subgroups relation of *P*,*D* and *G TPMS*. As mentioned in Table 7, *238 orbifold has a lower cost $|\chi_{o}|$ but is not triply periodic in $E^{3}$; adapted from [146].

The smallest possible genus for triply periodic minimal surfaces is g=3, and most classically known examples are in this class. The most symmetric one that we know is the *246 one that defines the *P*,*D* and *G TPMS* with a genus 3. Then, the lowest cost is $|\chi_{o}|$=1/24, much more than $|\chi_{o}|=1/60$ in $C_{60}$. A low $|\chi_{o}|$ value, for example, *237, breaks the translation symmetry in $E^{3}$. Subgroups of *246 orbifold give different *TPMS* with larger $|\chi_{o}|$, as depicted in Figure 28. A complete list of the 131 subgroups of *246 commensurate with *P*,*D* and *G TPMS* is given in [90].

#### 5.7.3. “Physical” Stability

Coming back to smooth surfaces, Willmore energy [145] is the mathematical view of the old problem of the elasticity theory. Willmore energy does not consider physical parameters such as “elasticity”. A more reliable functional was derived by Helfrich [145]. For a compact oriented surface *S* embedded in $R^{3}$,

(32) $$H\left(S\right)=a\int_{S}H^{2}dA+b\int_{S}KdA$$

where *a* and *b* are related to the flexural bending rigidity and bending stiffness, respectively [148]. Both parameters are related through the Poisson ratio ν [149]

(33) $$\nu =1+\frac{a}{b}$$

a=−b means that, when the material is compressed, it will not undergo any expansion. This is the case in Willmore model. The previous equation can be rewritten as

(34) $$H\left(S\right)=\int_{S}\left(2B_{M}H^{2}+B_{G}K\right)dA$$

where $B_{M}$ and $B_{G}$ are the bending rigidity and the Gaussian bending stiffness, respectively [148]. $B_{M}$ and $B_{G}$ are determined by *DFT* calculations: $B_{M}=1.44$ eV and $B_{G}=-1.52$ eV [148]. The surface occupied by an atom in the graphene lattice is $S_{0}=3\sqrt{3}d_{C-C}^{2}/4=3.63$ Å${}^{2}$

This model can be applied to nanotubes [148], fullerenes [148] and *TPMS* [22].

(35) $$E_{SWCNT}=E_{graphene}-S_{0}B_{M}R^{-2}/2$$

(36) $$E_{SWCNT}=E_{graphene}-1.9R^{-2}$$

R the radius in Å, energies in *eV*

(37) $$E_{fullerenes}=E_{graphene}-S_{0}\left(B_{M}+2B_{G}\right)R^{-2}$$

(38) $$E_{fullerenes}=E_{graphene}-3.58R^{-2}$$

In the same way, one defines the Willmore energy in *TPMS*.

(39) $$E_{TPMS}=E_{graphene}-1.52<R{>}^{-2}$$

**Figure 29:**
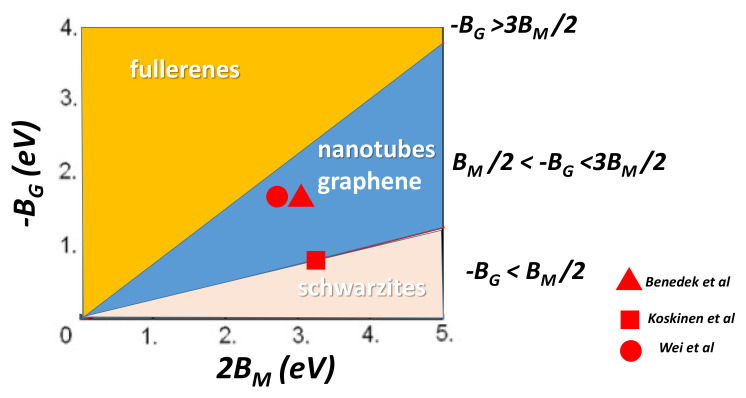
Domain of stability as a function of κ and $\bar{\kappa }$ (see text). The values were given by Benedek et al. [22], Koskinen et al. [150] and Wei et al. [148] (adapted from [22]).

<R> is the mean radius. In fact, the values of $B_{M}$ and $B_{G}$ are difficult to estimate. The topology of *GLC* is determined by the local values of both parameters, which are highly sensitive to the *t* parameter in Equation (15). Schwarzites are favoured when $-B_{G}<B_{M}/2$, nanotubes grow in the region $B_{M}/2<-B_{G}<3/2B_{M}$ and fullerenes $-B_{G}>3/2B_{M}$ (these relations are identical to those in [22] with $\kappa =2B_{M}$ and $\bar{\kappa }=-B_{G}$). Figure 29 displays the stability regions for the different families. Let us keep at mind that the transformation Schwarzite/fullerene or Schwarzite/nanotubes is unlikely for topological reasons: the genus is different and the curvature inversion needs the transformation of n-gons n>7 to n-gons n<6 with a prohibitive energy cost due to the bond breaking.

**Figure 30:**
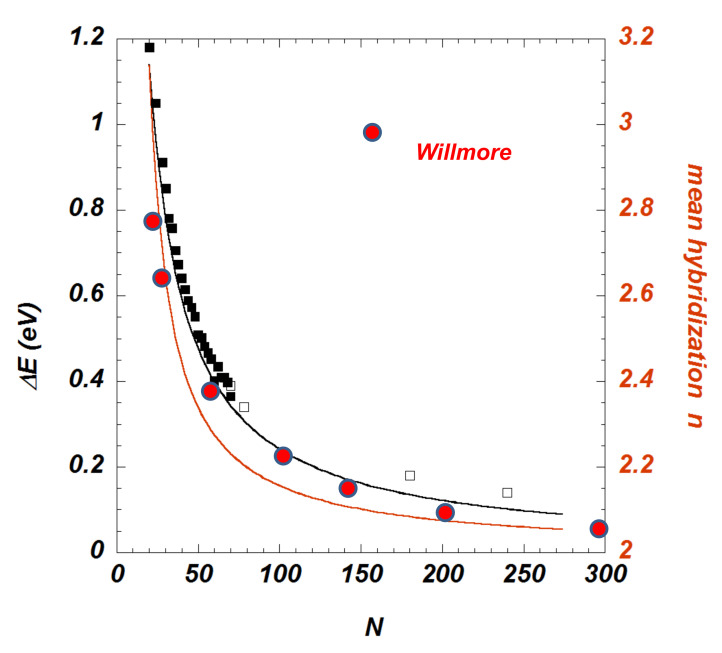
Relative energy to graphene in different models [153], *POAV* (bottom solid line) (Equation (42)), parameterised tight binding [154] (open square) and ab initio calculations [155] (filled square). For the latter, the energy is the difference between the total all electron local density functional density minus the total energy in the isolated atoms within the same formalism divided by the number of atoms. Since the authors do not report the value in graphene, a scaling factor has been taken by fitting the value in C${}_{60}$. The relative energy depends on the isomer under consideration. The hybridisation calculated from the POAV Equation (41) is also displayed. Values given by Equation (38) are also displayed.

This model can be compared to *POAV* framework (Figure 30). We recall that, in the π-orbital axis vector analysis (POAV) [54,151], the hybridisation is defined with geometrical consideration. Curvature (negative or positive) induces a misalignment of the orbitals (with respect to the planar graphene). A weak misalignment has several consequences [152]. First, the parallel alignment of p-orbitals is destroyed and the π bonding is less efficient. Weakening a π bond tends to raise the energy of the π bonding molecular orbital and lower the energy of the $\pi^{*}$ molecular orbital. A second consequence is the mixing between s-orbitals with the $p_{z}$-orbitals perpendicular to the basal plane that make up the primitive π system. Introducing the interorbital angle $\theta_{\pi \sigma }$, for fullerenes (N atoms) having a spherical shape [153],

(40) $$\sin\left(\theta_{\pi \sigma }-\frac{\pi }{2}\right)=\frac{2\pi^{1/2}N^{-1/2}}{3^{3/4}},$$

the mean hybridisation *n* is written as

(41) $$n=\frac{2}{1-\frac{4\pi }{\sqrt{3}N}}$$

and the energy to graphene is given by a simple relationship

(42) $$\Delta E\left(eV\right)=-3.1\times 10^{-3}{\left(\theta_{\pi \sigma }-\frac{\pi }{2}\right)}^{2}$$

### 5.8. *GLC* Modeling from *TPMS* Structure

#### 5.8.1. Constant Mean Curvature

**Figure 31:**
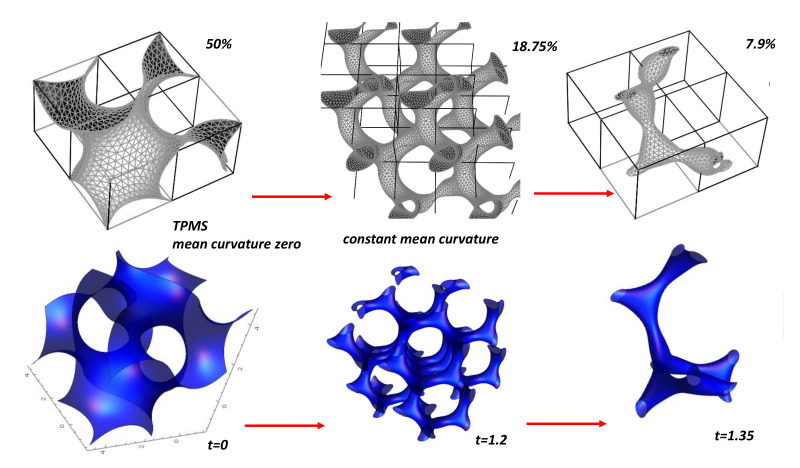
(**top**) *CMC* gyroid with various volume constraint deduced from Brakke’s Surface Evolver [156]. (**bottom**) CMC gyroid obtained with varying t parameter in Equation (15). (**top**) Reproduced with permission from Große-Brauckmann, Experimental Mathematic, [156]; copyright 1997, Taylor Francis.

**Figure 32:**
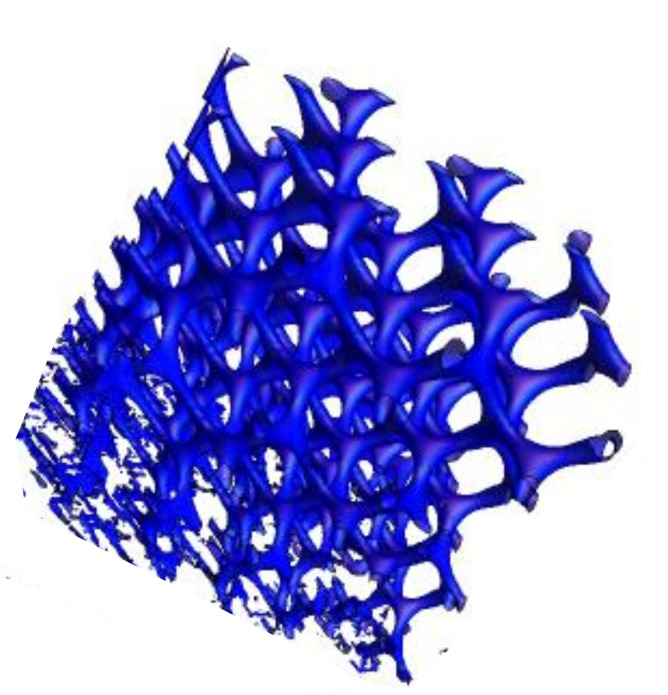
Random gyroid with β=0.4 and t=1.3 in Equation (44) with the parameters in reference [22].

Our starting point is the Barborini et al. model [14]. It is clear that gyroid *TPMS* in the standard form is far away the *GLC* structure. The first step is to modify the density. The problem is achieved by studying gyroids of Constant Mean Curvature (*CMC*). To do this, one considers the deformation of a minimal surface by embedding it into a continuous family of constant mean curvature surfaces [156,157]. Grosse-Brauckmann [156] used Brakke’s Surface Evolver to deform the *TPMS* keeping the same symmetry. Deformations of minimal surfaces are obtained by minimising the Willmore or bending energy under a volume constraint. The density is a key parameter. Figure 31 shows the case of a volume fraction of 18.75% compared to the minimal gyroid (50%). A simple approximation is obtained from Equation (15) adjusting *t* factor (t>0). The tubular form of the non-minimal *CMC* gyroid structure present striking similarities with an idealised *GLC* structure. Of course, *GLC* is not a crystal. Benedek et al. [22] introduced a randomisation of the trigonometric approximation given by Equation (15) (see Figure 32)

(43) $$\cos\left(x^{\prime }\right)\sin\left(y^{\prime }\right)+\cos\left(y^{\prime }\right)\sin\left(z^{\prime }\right)+\cos\left(z^{\prime }\right)\sin\left(x^{\prime }\right)=t{|}_{t=0}$$

where $x^{\prime }=f\left(x,y,z\right),x^{\prime }=g\left(x,y,z\right),z^{\prime }=h\left(x,y,z\right)$, *f,g,h* are defined functions. To take into a account the film growth anisotropy, the simple form was proposed [22] $x^{\prime }=xz^{-\beta }$, $y^{\prime }=yz^{-\beta }$ and $z^{\prime }=z^{1-\beta }/\left(1-\beta \right)$. All the trial functions must minimise the Osserman’s vector equation [158] giving the condition for a non-parametric minimal surface

(44) $$\left(1+\right|\frac{\partial f}{\partial y}{|}^{2}\frac{\partial^{2}f}{\partial z^{2}}-2\left(\frac{\partial f}{\partial z}.\frac{\partial f}{\partial y}\right)\frac{\partial^{2}f}{\partial y\partial z}+(1+|\frac{\partial f}{\partial z}{|}^{2})\frac{\partial^{2}f}{\partial y^{2}}$$

with in our case f≡x. Note that all the trial functions ($x^{\prime }=f\left(x,y,z\right)$…) except a linear scaling violate this equation. In other words, the complexity (randomisation) is paid for a lack of stability.

#### 5.8.2. Modified TPMS Structures: Strengths and Weaknesses

The simulation of the *GLC* structure by a mathematical analytic equation (after tessellation) is of prime importance to do calculations (*eDOS*, *pDOS*, etc.) Moreover, these structures (including randomisation) do not have any edges. This may explain the chemical robustness: the physi-chemisorption is on the dangling bonds of the edge atoms. Nevertheless, all the modified TPMS check the two congruent labyrinths and then are “unknot structures”. As mentioned previously, “unknot structure” is the best way for graphitisation (let us keep at mind that *GLC* are non-graphitisable structures). To summarise, random CMC are a good ground to model *GLC*. The low difference in cohesive energy between structures with positive curvature (fullerenes, onions, etc.) and negative curvature such as *TPMS* favours a mixing between them in *GLC* carbon. Random CMC trie to capture the essence of all the GLC properties but requires improvement

## 6. Conclusions

Thanks to the uniformisation theorem (geometry) and the classification one (topology), some amazing properties of *GLC* can be understood at first glance. The presence of *n*-gons with n>7 opens the door for the hyperbolic geometry and its fascinating world. Material properties are governed by mathematics and physics (or chemistry). Mathematics addresses the universal rules independently for the nature of bonding as long as the topology ignores the physical characteristics of atoms and atomic forces. It is interesting to separate universal properties related to mathematics and non-universal or “local” properties related to physics. The famous phrases “topological robustness” and “topological protected states” make sense. *GLC* and the entire $sp^{2}$ carbon family (or other *2D* compounds) are good candidates to do this. The possibility to synthesise *GLC* with well controlled conditions should allow in the future a better possible confrontation of problems thanks to topology. It remains a fascinating subject to study with many secrets yet to be discovered.

## Figures and Tables

**Table 1:** Selected properties observed in *GLC* compared to graphene. $I_{D}$ and $I_{G}$ refer to Raman spectroscopy. $d_{t}$ is the topological dimension.

Allotrope	$I_{G}$ Peak cm${}^{-1}$	*FWHM* cm${}^{-1}$	$I_{D}$ Peak cm${}^{-1}$	*FWHM* cm${}^{-1}$	*T* Peak cm${}^{-1}$
graphene	1582 ± 5 [25]	13	not relevant	not relevant	not relevant
*GLC*	1585–1600	25–(70)	1324–1329 [26]	40–(100)	weak 1150
**Allotrope**	**$I_{2D}$ Peak cm** ${}^{-1}$	**Density**	$d_{t}$	**Porosity**	**Plasmon π** [27] **eV**
graphene	asymmetric	not relevant	2	not relevant	6.4
*GLC*	symmetric	1.2–1.5 g/cm${}^{3}$	3	no	∼6
**Allotrope**	**Graphitisation**	**Conductivity**	**Chemical Stability**		
graphene	yes	semi-metal-like	good [28]		
GLC	no	complex [29,30]	very good [31]		

**Table 2:** Topological invariants for selected surfaces. The closed surfaces have no boundary β=0 (see Section 5.2). ω is the orientability number, which is 0 and 1 for orientable and non-orientable surfaces, respectively (see Section 5.1).

Surface	β	ω	χ	g
sphere	0	0	2	0
n-fold torus	0	0	2–2n	n
Klein bottle	0	1	0	
projective plane	0	1	1	
closed disc	1	0	1	
cylinder	2	0	0	1
Möbius band	1	1	0	2
closed orientable band	2	0	0	1

**Table 3:** χ calculated by algebraic geometry.

	*v*	*s*	*t*	χ
torus	1	−2	1	0
sphere	1	0	1	2
Klein bottle	1	1	0	0
projective plan	1	0	0	1

**Table 4:** Isometry (symmetry operator), orbifold symbol and associated Euler characteristic $\chi_{loc}^{i}$. All orbifolds contain a foundation sphere [70].

Isometry	Orbifold Symbol	$\chi_{\mathrm{loc}}^{i}$
(sphere)	1	2
pair of translations	o	-2
rotation centre	A	(1-A)/A
reflection line	*	-1
rotoreflection	(*) i	(1-i)/2i
glide line	x	-1

**Table 5:** Isometries of $E^{2}$ limited to the Coxeter class (for the definition, see  [74]). $\chi_{o}$ is the fractional Euler characteristic (see Section 5.7.2) [70].

Isometry	Orbifold Symbol	Group Number	$\chi_{o}$
*632	p6m	17	0
*333	p3m1	14	0
*442	p4m	11	0
*2222	pmm	6	0

**Table 6:** Isometries of $S^{2}$ limited to the Coxeter class. $\chi_{o}$ is the fractional Euler characteristic (see Section 5.7.2) [70].

Isometry	Orbifold Symbol	Group Number	$\chi_{o}$
*235	-	-	1/60
*234	m3m	221–230	1/24
*233	$\bar{4}$3m	215–220	1/12
*22k	-	-	1/2k
*226	6/mmm	191–194	1/12
*224	4/mmm	123–142	1/8
*223	$\bar{6}$2m	189	1/6
*222	mmm	47–74	1/4
*kk	-	-	1/k
*66	6mm	183	1/6
*44	4mm	99–110	1/4
*33	3m	156–161	1/3
*22	mm2	25–46	1/2
*	m	6–9	1

**Table 7:** Isometries of $H^{2}$ limited to the Coxeter class and $\chi_{o}>-1/12$. $\chi_{o}$ is the fractional Euler characteristic (see Section 5.7.2). Negative characteristics correspond to groups acting in the hyperbolic plane [70].

Orbifold Symbol	$\chi_{o}$
*237	−1/84
*238	−1/48
*245	−1/40
*239	−1/36
*23 (10)	−1/30
*23 (11)	−5/132
*23 (12), *246, *334	−1/24

**Table 8:** Crystallographic data corresponding to the three smallest cases of *D*, *P* and *G* Schwarzites [87]. *d* is the lattice parameter. The last line displays the gyroid illustrated in Figure 14. *D*688 indicates that he surface can be discretised by subdividing it into hexagons and octagons. Note that a gyroid on a smooth surface has a group I4132 (No. 214). Tessellation brings supplementary conditions with a Wyckoff position splitting for group–subgroup pair $Ia\bar{3}d$ (No. 230) > $I4_{1}32$ (No. 214).

Name	Space Group	d in Å	N	x	y	z
*D*688	$Pn\bar{3}m\left(224\right)$	6.148	24	1:2	0.33342	0.66658
*P*688	$Im\bar{3}m$ (229)	7.828	48	0.31952	0.31952	0.09373
*G*688	$Ia\bar{3}d\left(230\right)$	9.620	96	0.92205	0.12094	0.95502
gyroid	$Ia\bar{3}d$ (230)	18.599	384	0.18812	0.20968	0.77090
				0.07632	0.20151	0.84364
				0.02066	0.15594	0.87348

## Data Availability

The data in this study is available on reasonable request from the corresponding author.

## Appendix A

According to the Tyson course [159], we can define from a mathematical point of view the frontier between topology and geometry.

Let (X,d) and $\left(Y,d_{0}\right)$ be metric spaces (*X* is a set, while *d* is a metric on *X*) and let f:X→Y. Topology: *f* is a homeomorphism if *f* and $f^{-1}$ are both continuous without other conditions. Topological properties are unchanged under arbitrary homeomorphisms. This is the domain of the thermodynamic kinetics.

Bi-Lipschitz map: f is a bi-Lipschitz transformation if *f* and $f^{-1}$ are both Lipschitz, in other words if L<∞ is a number such that

(A1) $$\frac{1}{L}d\left(x,y\right)\leq d^{\prime }\left(f\left(x\right),f\left(y\right)\right)\leq d\left(x,y\right).$$

*d* is the distance d:X×X→R. Metric properties are unchanged under arbitrary bi-Lipschitz maps. This is the domain of the fractal world where *L* is the scaling factor.

Geometry: *f* is an isometry if *L = 1*. This is the domain of the standard crystallography.
